# Engaging with change: Information and communication technology professionals’ perspectives on change at the mid-point in the UK/EU Brexit process

**DOI:** 10.1371/journal.pone.0227089

**Published:** 2020-01-06

**Authors:** Elizabeth Lomas, Julie McLeod

**Affiliations:** 1 Department of Information Studies, iSchool, University College London, London, England, United Kingdom; 2 Department of Computer and Information Sciences, iSchool, Northumbria University, Newcastle-Upon-Tyne, England, United Kingdom; Politecnico di Milano, ITALY

## Abstract

**Background:**

Information and Communication Technology (ICT) has been a key agent of change in the 21^st^ century. Given the role of ICT in changing society, this research explores the responses and attitudes to change over time from ICT professionals and ICT academics in dealing with the potentially far reaching political challenge triggered by the UK’s 2016 European Union Referendum and its decision to leave the European Union (Brexit). Whilst the vote was a UK based decision its ramifications have global implications and as such the research was not confined to the UK. This article presents the second phase of the research at the mid-point in the UK/European Union (EU) Brexit process, thus complementing the findings gathered immediately after the Referendum decision. The fundamental question being researched was: What are ICT professionals’ personal and professional perspectives on the change triggered by Brexit in terms of opportunities and threats?

**Methods and findings:**

Data was collected through a survey launched in March 2018, one year on from the UK’s triggering of Article 50 and marking the mid-point in the two-year Brexit process. The survey replicated the one delivered at the point of the Referendum decision in 2016 with some developments. In addition, two appreciative inquiry focus groups were conducted. The research sought to understand any shifting perspectives on the opportunities and threats that would exist post-Brexit for ICT professionals and academics. 59% of survey participants were negative regarding the Brexit decision. Participants noted the position post-Brexit for the UK, and the remaining 27 EU Member States (EU27), was still very uncertain at this stage. They observed that planned change versus uncertainty provides for very different responses. In spite of the uncertainty, the participants were able to consider and advocate for potential opportunities although these were framed from national perspectives. The opportunities identified within the appreciative inquiry focus groups aligned to those recorded by survey participants with similar themes highlighted. However, the optimum conditions for change have yet to be reached as there is still not an informed position, message and clear leadership with detailed information for the ICT context. Further data will be gathered after the UK exit from the EU, assuming this occurs.

## Introduction

Information and Communication Technology (ICT) is fundamental to the delivery and support of all aspects of society, in addition to significantly contributing directly to each nation’s GDP [1, 2]. Over the last 50 years it has been a key agent of change [3, 4]. It has been seen to drive revolutions internationally and organizationally [5]. As such, those professionals who work in ICT and/or manage information, whether in the public or private sectors, are in theory well practised at harnessing opportunities in response to fast changing environments. ICT professionals have often been active participants in identifying, pushing and supporting change. Others involved in change processes may potentially be more passive actors in the transformation [6]. The focus of this research was to examine ICT professionals’ responses and attitudes to change through the lens of the challenges triggered by the Brexit Referendum decision, which has complex consequences. This event has potentially cast ICT professionals in a new role within the change process, whereby there is possibly less control but equally a need for ICT to contribute to and support the change as it emerges. The fundamental question being researched was: What are ICT professionals’ personal and professional perspectives on the change triggered by Brexit in terms of opportunities and threats? This provides for some learning about change and engagement with complex change more generally.

The Brexit decision is an unprecedented opportunity to chart a change process that will take a number of years and for which the overall outcomes were unclear at the time of the initial Referendum decision. For example, it was not known whether the Brexit decision would result in a clean UK break from the European Union (EU) or an altered relationship between the territories with some maintained UK-EU connections such as a customs union. Whilst Brexit is a UK decision, this shift in national relationships has potentially global ramifications. Aspects of ICT operate based on global innovation and network delivery. Therefore, this research sought to draw on global not just UK or EU perspectives.

This long-term study approached the data collection and analysis from a qualitative perspective in order to develop an understanding of attitudes to change through time. Qualitative studies do not require large data sets but rather analyze perspectives to enable an in-depth picture to emerge. This study used a STEEPLE model as a framework to structure data collection against seven factors: Socio-cultural, Technological, Economic, Environmental, Political, Legal and Ethical. Within an organizational context the STEEPLE model is used to understand the agencies of internal and external forces which influence change. It is a familiar tool for ICT professionals [7] [8].

The first phase of this research was a global survey launched on the first working day after the Brexit Referendum decision was announced in the UK (Monday 27^th^ June 2016), the results of which have been published [9]. This article presents results from the second phase of the research which was conducted between March and June 2018 through a global survey launched in March 2018 and two appreciative inquiry workshops in Newcastle and London. March 2018 was pertinent as it represents the mid-point within the period of notice provided by the UK to the EU prior to the UK’s theoretical exit from the EU on 29^th^ March 2019.

This article provides an analysis of the entire data set, including opportunities, threats and perspectives on responding to change from ICT professionals. It first provides a brief contextual background on ICT and change; this is followed by the key political events relating to Brexit following the UK Referendum decision in 2016 up until the end of July 2018 when the EU and UK Parliament broke for a summer recess, and finally a summary of key studies which deal with ICT considerations in relation to Brexit. The research methodology is then presented with a discussion of the data and findings. It is the intention to undertake a further survey after March 2019 (the planned date of a UK exit from the EU) in order to track perspectives on the change over time as events develop.

### Research context

#### ICT and change

ICT has been perceived to be an agent of change and revolution as it has enabled new ways of global communication, living and working. Bodrozic and Adler [5] highlight that as each technological revolution has occurred it has changed behaviors and models of working particularly in organizations. In addition, as discussed by Barrett et al [10], an increasing proportion of planned organizational interventions, designed to engender change, are managed through the active delivery of ICT. Within an organizational context the model of STEEPLE is used to understand the influences of internal and external forces on change considering seven factors: Socio-cultural, Technological, Economic, Environmental, Political, Legal and Ethical [7]. More et al [11] note the value of this approach to providing for strategic planning and organizational change. Whilst the STEEPLE model identifies influences on change it has often been aligned to change models which frame transitional steps. For example, Hiatt’s ADKAR Model [12] highlights a number of steps to change:

A—Awareness of the need for change which recognizes why change is necessary;
D—Desire to participate in and support the change;
K—Knowledge of how to change and what the change looks like;
A—Ability to implement the change on a day-to-day basis;
R—Reinforcement to sustain the change.

Many of the change models identify the emotional journey involved in change. For example, Kubler-Ross and Kessler’s model on human loss more generally has been shaped into a model used to understand responses to organizational or national change which take into account and acknowledge the loss, grief and ranging emotions involved in the change process [13]. Models by Kotter [14] and Bridge [15] respectively, deal with the same need to support individuals through the emotional process of change with each model focusing on moving the groups forward collectively taking into account that individuals have differing concerns and needs. In the model used by Kelly and Connor [16], information and communication help engender change as people can engage with and support a change even if they are pessimists, provided they are informed pessimists. Ultimately the goal is to move people towards informed optimism. Lawrence [17] identifies the process of participation as enabling this change and ending resistance. Appelbaum et al’s [18, p.215] synthesis of the change literature identifies different group dynamics which, whilst individuals have diverse engagement with the change process, nevertheless commit a group to moving towards a successful change. These are:

1. Affective commitment to change: beliefs in the inherent benefits of the change.
2. Normative commitment: sense of obligation to provide support for the change.
3. Continuance commitment: recognition of the costs associated with failure to support the change.

Key in change is strong leadership, collective dynamics and a clear and positive message around change crafted through time [19, 20, 21, 22, 23, 24 and 25]. In change contexts there will often be opportunities and threats; change is a process of risk management. Within an organizational context, case studies have indicated that change can be complex, with mixed outcomes and many projects failing [26]. In terms of political delivery there are very few government projects and initiatives which have been rigorously evaluated for their effectiveness in delivering largescale change. The closest comparison is in the history of warfare and diplomacy literature which deals with the influence of politics, power and negotiation in effecting change. In terms of ICT projects, governments have assessed delivering change through ICT transformation. Interestingly in this regard, a review of lessons learned from ICT delivery across national governments, concludes that projects fail when they are too complex and do not have embedded risk management processes including controls [27]. These issues are implicit in the Brexit context which is very complex and politically charged resulting in the risk planning not being open but rather confidentially held within the central UK and EU Government administrations. As noted in the risk management literature a lack of Brexit facts has created problems for planning [28]. Following the context of the politics of Brexit has been key to engaging with the change.

#### The politics of Brexit

On the 23^rd^ June 2016, the citizens of the UK (England, Northern Ireland, Scotland and Wales) and Gibraltar were given the opportunity to vote ‘leave’ or ‘remain’ to the single question ‘Should the United Kingdom remain a member of the European Union or leave the European Union?’ Following a voter turnout of 72%, 51.9% of the votes cast were in favor of leaving the EU [29]. Regionally the voter breakdown indicated that England and Wales were in favor of leaving the EU with Gibraltar, Scotland and Northern Ireland in favor of remaining. These regional differences have been significant in the subsequent discussions and political perspectives on the shape of any potential agreement between the EU and the UK and Gibraltar. Ownership of voting ‘leave’ has been complex with some voters reporting a stigma attached to voting leave and others indicating that their vote was a protest vote made on the assumption that the ‘leave’ campaigners would not win [30, 31]. There has also been conflict across the age spectrum with older voters being blamed for the leave vote and a suggestion that with younger voters becoming eligible to vote this would make a difference if there were a second vote, although typically the election turnout for younger voters is lower [32].

The process of the UK exiting the EU follows certain key stages. In the first instance the UK provided notice to the EU of its intention to leave by triggering Article 50. Following this trigger, the UK agreed with the remaining 27 EU Members States (EU27) a political mandate for the negotiations after which the negotiations commenced [33]. Table 1 below provides a high-level overview of key political events following the Brexit Referendum decision up until the end of July 2018.

**Table 1:** Timeline of key political Brexit related events 23 June 2016–31 July 2018.

Date	Event
23 Jun 2016	UK Referendum on whether to ‘remain’ or ‘leave’ the EU.
24 Jun 2016	Decision of the UK Referendum vote to leave the EU is announced with 51.95% of votes cast in favor of ‘leave’. The UK Prime Minister (PM) David Cameron (Conservative Party) resigned from office.
13 Jul 2016	In the UK Theresa May selected by the Conservative Party as their leader and thus the new UK PM.
14 Jul 2016	The UK established the Department for Exiting the European Union. David Davis was appointed the Secretary for Exiting the European Union.
27 Jul 2016	EU President Jean Claude Juncker appointed Michel Barnier as the EU’s Chief Negotiator in charge of the preparation and conduct of the negotiations with the UK under Article 50 of the Treaty on the European Union.
2 Oct 2016	UK PM Theresa May announced in front of her party the Conservatives at their annual conference her plans for Brexit including the delivery of a Great Repeal Bill (later known as the European Union (Withdrawal) Bill), which she stated would not require UK Parliamentary approval.
3 Nov 2016	Gina Miller, together with London-based Spanish hairdresser Deir Tozetti Dos Santos and the People's Challenge group, launched a challenge of the UK Government’s right to trigger Brexit without Parliamentary approval. The case of *R (Miller and Dos Santos) v Secretary of State for Exiting the European Union* became known colloquially as the ‘Miller’ case. On 3 November 2016, a High Court Judgement found in favor of ‘Miller’. The UK Government immediately confirmed it would appeal to the Supreme Court.
7 Dec 2016	UK and Gibraltar Government met for first talks set up under the Joint Ministerial Council (Gibraltar EU Negotiations).
17 Jan 2017	UK PM Theresa May set out her ‘Plan for Britain’ in the light of Brexit at a speech made at Lancaster House, London. This included 12 negotiating priorities.
24 Jan 2017	The UK Supreme Court rejected the UK Government’s appeal against the ‘Gina Miller’ case upholding the decision of the High Court that only the UK Parliament could approve the Brexit process.
28 Jan 2017	European Union (Notification of Withdrawal) Bill introduced to UK Parliament. This was designed to repeal the UK European Communities Act 1972.
2 Feb 2017	UK PM Theresa May’s Brexit White Paper introduced to Parliament ‘The United Kingdom’s exit from and new partnership with the European Union’.
16 Mar 2017	European Union (Notification of Withdrawal) Act received Royal Assent in the UK Parliament.
29 Mar 2017	UK PM Theresa May wrote to European Council President Donald Tusk triggering Article 50 of the Treaty of the European Union which thus provided notice that the UK would withdraw from the EU in two years’ time.
30 Mar 2017	Great Repeal Bill White Paper introduced into Parliament–foundation legislation for the UK’s withdrawal from the EU. Scotland and Wales referred to this as a ‘power grab’ as the paper proposed powers from the EU would pass back to the UK Parliament rather than the devolved administrations.
18 Apr 2017	UK PM Theresa May announced a UK General Election on grounds of requiring ‘stability and certainty’ to deliver the Brexit process.
23 Apr and 7 May 2017	The French Presidential Election was held with Emmanuel Macron, as leader of ‘La République *En Marche*!’, receiving 66% of the vote in the second round of the Election process defeating Marine le Pen who was fighting a right-wing nationalist campaign and had spoken in support of the UK’s Brexit decision.
9 Jun 2017	At the UK General Election PM Theresa May’s Conservative party lost its majority winning only 318 seats from 650 seats. A UK hung Parliament resulted.
26 Jun 2017	The Conservative Party and the Democratic Unionist Party (DUP), a Northern Irish party, signed a ‘Confidence and supply agreement’ which provided the Conservative Party with the required support to form a UK Government.
22 Sep 2017	PM Theresa May met with President Jean Claude Barnier to further set out the UK position on Brexit in her so called ‘Florence speech’.
23 Sep 2018	German Federal Election was held in which the Christian Democratic Union received the largest proportion of votes although the percentage of the Christian Democratic Union vote was diminished. As such, Angela Merkel retained the position of Chancellor but was weakened.
9 Oct 2017	Liam Fox, UK Secretary of State for International Trade and President of the Board of Trade, introduced ‘Preparing for our future UK trade policy’ in Parliament.
4 Dec 2017	Talks were held on Brexit between the EU and UK. The Republic of Ireland PM and wider EU membership indicated hopes that Northern Ireland could have a special status in the Brexit process in order to avoid the need for a border. However, the DUP announced it could not agree circumstances for a Brexit which might undermine the basis of the UK Union. This stance was deemed to have ‘derailed’ the negotiations.
8 Dec 2017	UK PM Theresa May and President of the European Commission Jean Claude Junker held a joint press conference signaling a way forward for the Brexit negotiations. The DUP signaled approval for these statements.
15 Dec 2017	The EU27 agreed to move to phase two of the Brexit process. This indicated an acceptance that negotiations would move on to discuss a transition period and the future relationship between the EU and the UK.
17 Jan 2018	Welsh Assembly Members voted unanimously in support of a Plaid Cymru motion calling for a new law to defend Wales from UK Brexit plans.
29 Jan 2018	Supplementing negotiating directives for the Brexit negotiations were produced, which detailed the EU27 position regarding a** transition period**. These negotiating directives provided the Commission, as the EU negotiator, with a mandate to start discussions with the UK on this matter.
6–9 Feb 2018	EU27 and UK Article 50 negotiations held in Brussels. Leaked UK Government analysis of the impact of Brexit on trade suggested free trade deals with non-EU countries would add less than 1% to long term UK economic growth.
23 Feb	EU27 and UK discussed post-Brexit EU budget.
26–27 Feb 2018	EU27 and UK Article 50 negotiations held in Brussels.
27 Feb 2018	The Scottish Government introduced the UK Withdrawal from the European Union (Legal Continuity) (Scotland) Bill.
28 Feb 2018	European Commission published a draft ‘Withdrawal agreement on the withdrawal of the United Kingdom of Great Britain and Northern Ireland from the European Union and the European Atomic Energy Community’. This set out three options for the Irish border: Option A—a future relationship between the UK and the EU that avoids the need for a border; Option B—unique solutions—including technology—be found to solve the issue; Option C—that Northern Ireland remains aligned with EU rules in a number of areas.
16–19 Mar 2018	Further talks were held between UK Secretary for Exiting the European Union David Davis, and the EU Chief Negotiator for Brexit Michel Barnier. The EU published a further draft agreement on the withdrawal of the UK from the EU. The agreement set out: EU citizens arriving in the UK between the withdrawal dates would enjoy the same rights and guarantees as those who arrived before Brexit. The same rule would apply to UK expats on the continent; the UK would be able to negotiate, sign and ratify its own trade deals during the transition period; the UK would still be party to existing EU trade deals with other countries; the UK's share of fishing catch would be guaranteed during transition but UK would effectively remain part of the Common Fisheries Policy, yet without a direct say in its rules, until the end of 2020; Northern Ireland would effectively stay in parts of the single market and the EU Customs Union in the absence of other solutions to avoid a hard border with the Republic of Ireland.
21 Mar 2018	The Welsh Assembly introduced the Law Derived from the European Union (Wales) Act 2018.
22–23 Mar 2018	At an EU Summit the Agreement on the withdrawal of the UK from the EU was passed and signed off in two minutes.
Apr 2018	Formal talks continued in Brussels particularly on the issue of the border between the Republic of Ireland and Northern Ireland and what would happen after the UK's withdrawal from the European Union to 64 powers in devolved areas, such as agricultural support and food labelling.
15 Apr 2018	‘People’s Vote Campaign’ launched calling for a second Referendum for the UK on the final EU deal.
17 Apr 2018	The Attorney General for England and Wales and the Advocate General for Scotland referred to the Supreme Court the EU exit legislation introduced by the Welsh Assembly and the Scottish Parliament to determine whether these laws were constitutionally within the devolved legislative powers.
7 Jun 2018	PM Theresa May revealed the so called ‘Irish back stop plan’ which would provide for a temporary customs arrangement for up to a further year to ensure the avoidance of a hard border between the Republic of Ireland and Northern Ireland.
26 Jun 2018	European Union (Withdrawal) Act 2018 passed into UK law.
28–29 Jun 2018	EU-UK Summit Meeting
31 Jun 2018	EU President Jean Claude Juncker addressed a joint sitting of the Dáil and Seanad (the two Houses of the *Oireachtas *the Parliament of Ireland) and acknowledged the potential of a no deal Brexit. Junker stated that Ireland would come first in negotiations. This promise related to concerns that a hard border would be drawn between the Republic of Ireland and Northern Ireland.
6 Jul 2018	The UK’s Cabinet met at Chequers to set out a UK vision for Brexit. This became known as the ‘Chequers Deal’. Included in the deal was the concept of a Common Rule for all goods with a commitment to harmonization with EU rules where necessary.
8–9 Jul 2018	The UK’s Secretary of State for Exiting the European Union (Brexit Secretary) David Davis resigned and was replaced the next day by Dominic Raab. Other resignations followed including the Foreign Secretary Boris Johnson.
12 Jul 2018	UK Government published a policy paper ‘The future relationship between the United Kingdom and the European Union’. This set out an agenda for the UK EU relationship including a ‘Common Rule book’.
16–17 Jul 2018	House of Commons, UK Parliament Customs Union Vote won by Government.

Since the Brexit vote, within the UK, politicians, media, wider commentators and citizens have remained conflicted on what the Referendum decision means in reality in terms of its delivery. Commentators have argued that the simple binary choice of leaving or remaining within the EU meant the vote was not constructed to properly surface citizens’ wishes in terms of the shape of the future relationship between the UK and EU. Within the UK Parliament, MPs have remained split across all parties regarding stances to Brexit. Whilst the immediate impact of the decision did not bring widespread chaos to the UK, as some had predicted, there are a range of views on the longer terms implications of leaving depending on the terms of departure from a range of perspectives including the position for services, goods and agriculture. The idea of the UK leaving the EU in March 2019 with no deal in place has been claimed by many politicians and economists to be the worst potential outcome for UK business. However, a key issue in the discussions has been the importance of recognizing the democratic decision and voice of citizens in contrast to evaluating the economic damage for the UK if leaving the EU is fully enacted as a ‘hard Brexit’ which would mean that there would be no ongoing customs union [34]. In Europe there have been some calls within other nations for discussions on exiting the EU, e.g. France/Frexit, Italy/Italexit and Poland/Polexit, but to date these have not gained mass support. The EU27 have seemingly presented a united negotiating stance in dealing with the UK. Brexit developments have received ongoing global news coverage and comment.

Within the UK, YouGov polls [35, 36] have charted citizens’ perspectives on Brexit. These evidence an overall shift in terms of perspectives on the potential impact of Brexit with some fluctuations. For example, in the sample polled on whether the UK would be economically worse off following Brexit, 35% agreed that it would be in 2016 and 40% in 2017. 22% felt it would be better off in 2016 and 28% in 2017. The polls clearly evidence the continuing polarized perspectives from the Leavers and Remainers. In 2017, 78% of Remainers felt the UK would be economically worse off and only 7% of Leavers. However, what is likely to be the overall outcome of the Brexit decision is perceived by citizens to be complex. On 29^th^ June 2018, Danny Dyer a UK celebrity, summed up the national mood of many by describing Brexit as “No one knows what it is–it’s like this mad riddle that no one knows what it is, right” [37]. The Brexit situation is somewhat akin to the political context Donald Rumsfeld described in regard to the global war on terror in 2002:

The message is that there are no "knowns." There are things we know that we know. There are known unknowns. That is to say there are things that we now know we don't know. But there are also unknown unknowns. There are things we don't know we don't know. So when we do the best we can and we pull all this information together, and we then say well that's basically what we see as the situation, that is really only the known knowns and the known unknowns. And each year, we discover a few more of those unknown unknowns. [38]

In this regard, the context of the lens of Brexit as a focus for understanding change has to date provided a situation where the stages of change are not currently clearly defined.

#### Brexit and ICT

If the UK leaves the EU without a deal with the EU, then it becomes a ‘third country’ from the perspective of the EU. This has implications for the ICT sector in terms of supply chains and tariffs, which may apply for the transport of goods. It will increase the cost of movement of goods between the UK and the rest of Europe. The World Trade Organization has a list of the taxes which will apply to goods outside of trade agreements which make evident the economic implications. In addition, the accompanying paperwork requirements will slow up operations between the regions. Separately there are implications for ICT service impacts, which are harder to ascertain as services are supplied in more complex ways; for example sometimes in person or sometimes through channels with more complex borders. Whilst services are not restricted by borders and tariff barriers in quite the same way as goods, nevertheless other barriers may exist. There may be licensing, quotas, or professional qualifications, which determine whether foreign providers are allowed to enter a market. In addition, the legislative landscape for operations shifts, if the UK leaves, as information rights laws are impacted. For example, the EU would need to determine whether the UK had appropriate safeguards in place relating to the protection of personal data, i.e. GDPR ‘adequacy’, in order for the UK and EU to continue to exchange personal data. For intellectual property law and trademarks, an EU trademark will no longer cover the UK, so organizations would need to take steps for UK protection. Other laws around intellectual property will require similar evaluation, including software development and copyright. These considerations have implications, as the ICT industry will consider physical operational logistics weighed against differing concerns related to services and customer bases.

In determining the impact of Brexit and the permutations for a deal, the UK Government has taken evidence from a range of experts and sectors. The July 2017 *Technology ICT Sector Report*, produced by the UK House of Commons Committee on Exiting the European Union, recognized the role of ICT in supporting the UK’s delivery of services and goods, providing employment and income generation [39]. This picture has been further developed by the evidence provided to the UK House of Commons Digital Culture and Media Sport Committee as published in January 2018 [40]. Other important work has been undertaken to understand aspects of ICT delivery which are not necessarily specific to the Brexit situation but have helped to develop the picture, e.g. the ScotlandIS report on ICT public sector expenditure in Scotland [41].

In an EU context there have been discussions at Committee level on the impact of Brexit on ICT policies. For example, in the context of the Industry, Research and Energy Committee, these have revolved around the implications for policymaking and regulation, as well as innovation and deployment of 5G [42]. A testimony by J Scott Marcus, before the Committee in June 2018, warned that a complete break between the UK and EU was not advisable as it would have a significant impact, for example in the Artificial Intelligence (AI) research and development arena [43]. Key for the UK in terms of the support for innovation has been the EU research relationship and funding which remains to be determined [44].

In a trading context there has been widespread discussion of the potential impact. In 2015 Nesta launched a European Digital City index which, in November 2016 after the Referendum vote, still placed London first out of 60 European cities as a location for digital startups with eight other UK cities featuring in the listing [45]. However, interviews and analysis by Oliver Patel, UCL, in December 2016 indicated that across the ICT sector there was concern regarding immigration status for those working in this sector in the UK and, in addition, regarding future access to skills as well as market access for UK businesses [46]. In 2017, Richard Reynold from Atradius commented that whilst the Brexit decision will impact the UK ICT sector, particularly given the significance of EU markets, there remain sector opportunities [47]. Atradius, in 2018, indicated that the UK ICT industry had been worth 97 billion GBP to the economy in 2017, which was up 30% from 2012, but that due to Brexit uncertainties this growth had slowed, in part because of concerns about talent shortages [48]. However, considering the significance of the ICT sector to the UK and EU there has been limited information and analysis put into the public domain in terms of the impacts to ICT goods and services.

## Methods

The overarching aim of this research was to gather an in-depth picture of ICT professionals’ responses and attitudes to change through the lens of the challenges triggered by the Brexit Referendum decision. The fundamental question being researched was: What are ICT professionals’ personal and professional perspectives on the change triggered by Brexit in terms of opportunities and threats? The UK’s Brexit Referendum decision represented a major exogenous shock for the ICT industry, which would require responses and actions through time. Whilst its impact was unknown at the outset, it was envisaged that the changes required would become evident more quickly than has potentially been the case.

The study took a qualitative approach. Qualitative research was determined to be best suited to the research aim. It enabled an inductive approach to the study, which was important for considering change with unknown outcomes. In addition, the particular strengths of qualitative research are that it can provide insights into complex events where there are many interconnecting factors some of which are intangible considerations that are difficult to identify and measure quantitatively [49, 50]. As noted by Dafoe and Lyall, rich causal processes relating to ICT and human dimensions, can have varied, complex dynamics which quantitative research may fail to identify [51]. In the context of Brexit, there were many complex factors and tensions between personal, professional, national and industry specific considerations. In an ICT context, different considerations exist dependent upon the nature of the service delivery, for example whether there are goods or services involved in a supply chain. The data was collected via a survey and appreciative inquiry focus groups. The former provided an opportunity for fully anonymized free and frank exchanges. The latter enabled collaborative discussions to develop and counter key considerations.

The research was a collaboration between University College London and Northumbria University. It went through the University College London Research Committee including providing data protection approval (Ref: UCL/13101/001).

### Survey

The survey (S1 Fig) was developed and administered using the online survey tool Opinio which is UK hosted for EU data protection purposes. The survey was open to anyone across the world. Whilst Brexit itself was an emotive issue, contributions were requested to be made in a constructive manner. It was noted by a number of potential participants working in industry contexts, and in particular UK based banking, that for cyber security reasons they were not allowed to click on links to online surveys. As such this potentially limited the pool of contributors.

The intention was to repeat the 2016 survey, in order to be able to consider changing perspectives. The participants were not necessarily the same although the question was asked whether individuals had previously participated. Some alterations were made to the survey design to take into account considerations identified from the previous data set. For this second survey it was possible to plan the work over a longer timeframe and gain ethical approval to ask further questions. In addition to collecting the participants’ place of residence and nationality, their age and gender were collected. Some studies have indicated that age and gender influence attitudes to change and that this can be a significant variable [52]. In line with the previous survey, in order to aid the protection of the identities of those who participated, IP address data was not captured. The survey provided an opening consent page which explained its purpose. All questions were optional. In order to enable free and frank contributions permission was not sought for the full data set to be shared as anonymity was promised. All the qualitative data quoted was carefully manually reviewed in order to protect both personal data and organizations’ commercial confidentiality.

In line with the 2016 survey, a STEEPLE model was used as the central data collection framework, which is a common tool used to understand agents of change in an ICT context [7, 11]. The STEEPLE model provided participants with a familiar framework of prompts to consider the opportunities and threats against seven factors: Socio-cultural, Technological, Economic, Environmental, Political, Legal and Ethical. Participants from the 2016 survey had commented that on occasions it was problematic to separate out these factors and furthermore they had sometimes wanted to discuss their answers making connections across the factors. Taking this into account, the second survey asked participants to respond to the opportunities with reference to any or all of the factors, rather than each one individually, and then to repeat this process considering the threats. This enabled participants to respond in one text box highlighting observations and linking these on occasion to multiple factors. As the survey was trying to understand ICT change in the light of Brexit, participants were asked whether they had completed the first survey, whether they had changed their attitudes on Brexit since the Referendum, and whether, as an ICT professional, they felt confident in dealing with uncertainty.

The possibility of translating the survey into French and German was investigated in order to gain a wider set of EU responses. However, funding did not allow for translation assistance and therefore it was ruled out for this survey iteration. The fact the survey was only in English did potentially limit the pool of participants.

The survey was piloted on five researchers and five practitioners from the target audience. It was distributed globally by the authors using a virtual snowballing (i.e. referral) sampling method [53]. The initial launch was at the opening of the international iConference on Sunday 25^th^ March 2018 in Sheffield, UK. This conference attracts ICT academics and some professionals. Contacts at the conference were asked to tweet the survey link which initiated the snowballing process. At the same time messages were posted through direct email, email listservs, online newsletters and social media posts (e.g. Facebook, LinkedIn and Twitter). The survey remained open until 27^th^ April 2018. This chosen date aligned to the mid-point in the planned two-year period for negotiations. It is to be noted that this also coincided with a holiday period in many countries and the researchers did receive some comments that potential participants would have completed the survey if it had been left open for longer.

During the period of data collection, research participants could amend or add to their answers. The survey data was analyzed manually and using Excel. An inductive process to coding the qualitative responses was adopted, utilizing the steps set out by Lewins and Silver [54]. Codes were not reviewed from the first survey in order to try to minimize bias in the coding. Line by line open coding was undertaken and axial codes were then developed to identify linked themes. The data was then reviewed and under each coded axial theme every separate mention of an idea identified against that theme was counted under the relevant STEEPLE factor. Participants had largely self identified the relationship to the STEEPLE factors. The total count of all mentions across the STEEPLE factors was then tallied. The process of counting in qualitative approaches is recognized to be controversial as each single contribution by a participant has value. It is recognized that qualitative coding is a subjective sense making process. Counting can help make sense of data when dealing with largescale complex issues but is nevertheless potentially contentious [55]. In order to address these issues, the full list of open codes has been provided in addition to the axial codes or themes (S1 Table). This has been checked to ensure that the coding provided does not reveal any personal or commercially confidential data.

### Appreciative inquiry focus groups

In addition, two appreciative inquiry workshops were held, the first in Newcastle on 3^rd^ May 2018 and the second in London on 26^th^ June 2018. At each workshop there were 9 participants. The participants included both UK and international representatives. Dynamo North East (http://www.dynamonortheast.co.uk/), a community interest company whose mission is to grow the IT economy in the North East of England, assisted with sourcing participants for the Newcastle event. Each of the participants was provided with an information sheet and consent sheet (S2 Fig). Participants could choose to be acknowledged for their participation or to have their contributions anonymized.

Appreciative inquiry is a strengths-based approach, intended to focus only on the potential positives for change and to develop these possibilities [56]. It seeks to establish a basis for positive development and change. In the shared setting of a focus group, positive discussions can be developed, whereas the challenging situation of lone contemplation can make positivity difficult. In the context of the challenge posed by Brexit, this more positive collaborative approach enabled all participants to contribute to analyzing IT perspectives and possibilities regardless of their personal perspectives on the Brexit Referendum decision. The participants were asked to assume that Brexit would occur, although it was acknowledged at the outset that the format it could take and the continuing relationships between the UK and other EU member states were uncertain.

Within each workshop participants were split into two sub-groups and, as in the survey, the STEEPLE model provided the framework for focusing their discussion (S3 Fig). The sub-groups then rejoined and discussed their perspectives on each of the seven factors. They then concluded the workshop by pulling out their highlight opportunities. Responses were not coded and counted but rather themes were captured on flipchart sheets. The responses in this context reflected a rich set of shared narratives.

### Participant demographics

A total of 245 participants completed the survey. Despite cascading the survey through the same networks as for the first survey, only 27 participants (11%) confirmed that they had completed the first survey. Table 2 indicates the gender, age (S2 Table), nationalities (S3 Table) and locations (S4 Table) of those who responded and compares these to 2016 responses. Table 3 contains the professional contexts for the participants. The modal values for each variable are highlighted in grey in Tables 2 and 3. Whilst less people responded to this second survey, they indicate a relatively similar statistical profile of the background demographics and contexts of the participants.

**Table 2:** Personal characteristics of survey participants.

	2016 Survey	2018 Survey		2016 Survey	2018 Survey
**Age**	**%**	**%**	**Nationality**	**%**	**%**
18–24	1.5%	2.5%	UK	59.5%	59%
25–34	20%	15.5%	EU excluding UK	25.5%	10%
35–44	30.5%	17%	Rest of World	6%	11%
45–54	27%	22.5%	Other	9%	20%
55–64	17%	14.5%	**Place of residence**	**%**	**%**
65–74	2%	1.5%	UK	75%	64%
75+	0.5%	0.5%	EU excluding UK	18.5%	7.5%
Prefer not to say	1.5%	26%	Rest of World	5.5%	8.5%
**Gender**	No Data Collected	%	Other	1%	20%
Male		40%	Modal values shaded in grey		
Female		32%	All percentages are rounded to the nearest 0.5%		
Other/prefer not to say		28%			
	

**Table 3:** Professional contexts of survey participants.

	2016 Survey	2018 Survey		2016 Survey	2018 Survey
Profession/job role–participants sometimes noted multiple roles	%	%	Number of employees in organization where participants were employed/volunteered	%	%
Academic	14%	15.5%	0–9	7%	11%
Administrator	2%	4%	10–49	8%	9%
Archivist	15%	15%	50–249	14%	10%
Business analyst	1%	1%	250–999	20%	25%
Cyber security/Information security	3%	4.5%	1000–9999	41%	36%
			10000+	10%	8%
Data manager	3%	1.5%	Prefer not to say	0%	1%
Information manager	18%	12%	**Global organization**	**%**	**%**
IT support	3%	2.5%	Yes	28%	35%
IT project manager	12%	3.5%	No	72%	65%
Lawyer	1%	0.5%	**Occupational status**	**%**	**%**
Librarian	24%	19.5%	Employee	86%	76.5%
Management consultant	1%	3%	Self-employed	5%	10.5%
			Unemployed	1%	0.5%
Managing Director	0%	2.5%	Student	5%	6%
Marketing manager	1%	1.5%	Volunteer	1%	3%
Mergers & Acquisitions	0.5%		Retired	1%	2%
Records manager	10%	6%	Other	1%	1.5%
Software developer	2%	0.5%	Modal values shaded in grey		
Telecoms manager	0.5%	0%	All percentages are rounded to the nearest 0.5%		
Web designer	1%	1%			
Other	2%	30%			
**Management role**	**%**	**%**			
Management role	48%	49%			
No management role	51%	51%			
N/A	1%	0%			

In respect of personal demographics, a number of participants noted their complex nationalities and residency statuses or did not wish to comment on these. 40% of the survey participants indicated they were female, 32% male with 28% preferring not to comment. Three people explicitly commented that questions on so called ‘gender’ were no longer valid. There were very low numbers of responses from those at either end of the age spectrum, which is potentially to be expected for a professional survey. 26% of participants did not wish to state their age, with a lower percentage of participants from 45 years upwards identifying as completing the 2018 survey than in the 2016 survey. This result may be due to the conflict since the Referendum around voting patterns linked to age, with younger voters condemning older voters for voting ‘leave’. It potentially evidences the 2018 survey participants’ sensitivities to wider social divisions within the UK caused by the Referendum decision.

Despite being circulated globally, the majority (64%) of participants were based in the UK as in the 2016 survey. 59% of participants were UK nationals. The highest number of European responses, excluding UK nationals, was from the Republic of Ireland (3.5%) followed by France (2.5%). Excluding EU nationals, the highest number of responses was from the USA (3%). Only one person responded from Belgium although it is at the heart of EU administration. 20% of participants either preferred not to specify their location or nationality or commented that this was complex. Several participants were in the process of transitioning to new locations due to the Brexit decision. In the 2018 survey, there were less non-UK EU participants than in the 2016 survey although the matter has been widely debated across the EU news. There was a 15.5% drop in non-UK EU participants. However, there was an 11% rise in participants choosing to identify complex nationalities or specifically indicating they did not wish to state their nationality. The complexity of nationality has been brought to the fore by Brexit, in circumstances where people are now needing to make passport and residency choices. In terms of residency, the participant figures also shifted with a drop of 11% respectively both in terms of the participants resident in the UK and in the EU excluding the UK. 19% of people indicated complex residency or preferred not to state their place of residence.

In respect of professional demographics, academics from ICT related disciplines and librarians provided high numbers of responses in both the 2016 and 2018 survey. However, the 2018 survey captured responses from a wider range of participant professions. Several participants noted their connection to multiple professions and job roles. In both surveys, 51% were not managers with 48%/49% in management roles. 35% were working or volunteering in global enterprises, with many others noting in the comments fields that their organizations, whilst located in one country, managed information, markets and services across geographical boundaries. The number of participants from global organizations rose by 7% with an increase of 7% of participants being from organizations with over 1000 employees. Potentially global organizations have been engaging more actively with Brexit planning. The majority of participants in 2016 and 2018 were employees.

Within the workshops three participants were resident outside the UK and 15 were UK based. 14 of the participants were working in organizations or roles with international networks or bases. The participants who chose to be acknowledged were Sally Edgar, Adam Hill, May Ladd, Professor Tero Päivärinta, Cesare Rizzo, Alan Shipman and Professor Hrvoje Stancic.

### The Brexit context

Despite the potential wider impact of the Brexit decision and global media coverage, it is important to note that the survey was largely completed by UK citizens. This is potentially telling in terms of how significant other nationalities perceive this issue. However, even though Brexit is of significance for the UK, a number of UK contacts did indicate they were ‘weary of the topic’ or ‘depressed by this’ and did not feel motivated to spend time on the survey. This emotion was also expressed within some of the actual participant survey responses:

“Tired of it all—it is difficult to feel motivated by any of it.”

In the context of the workshops, three out of four international participants approached agreed to attend; one declined due to commitments elsewhere. Only one out of nine participants approached in the UK agreed to attend the workshops with the reasons for declining including commitments elsewhere, but also either a sense that it was problematic to contribute whilst the situation was still so uncertain or often weariness for the topic. In addition, UK participants were not keen to be associated with a Brexit event as, despite being able to anonymize their contributions in publications, some felt it would be harder to guarantee complete anonymity given the group dynamic. Given that workshop participants potentially felt less able to be anonymous, they were not asked for their age, gender or voting position on Brexit, only their national and industry contexts. Many workshop participants chose to disclose their position on the Brexit vote at the outset of this event largely in many cases to dismiss any assumptions that they were pro-Brexit, although some participants were ‘leave’ supporters.

This problem of acknowledging a ‘leave’ vote is potentially evident in the survey where it was of note that on a number of questions relating to perspectives or a voting position on Brexit, people did not wish to comment on their position despite the assurance of anonymity. ICT professionals are predominantly situated in the demographic of remain voters, as indicated in the 2016 survey results. Therefore, voting leave is potentially more contentious to declare professionally.

## Results and discussion

### Plans post Brexit

In terms of organizational contexts, 18% of the 2018 survey participants indicated that there were no plans in place relating to the Brexit decision, 28% of participants did not know if there were plans in place and 28% indicated that there were now plans in place within their organization in the light of Brexit. This was a significant increase from the position in June 2016 where only 6.5% of participants indicated that their organization had any plans. However, it is notable that nevertheless it remains a relatively low percentage given that the Referendum decision had now been known for some time. As one person commented from a UK perspective, “there seems to be a seemingly false hope we will not leave”. For those who had plans in process these were wide ranging, with some indicating working groups/committees actively meeting to manage potential change. A number of participants commented that their organization was looking at new locations and relocating parts of the business/organization, mapping new supply chains/agents, leveraging currency fluctuations, identifying areas of future growth including international global consultancy outside the EU, reviewing regulatory change, identifying wide-ranging needs, fostering UK/EU links, and lobbying the UK Government for a deal supporting businesses. In addition, there were a high number of comments relating to protecting and supporting staff with different nationalities. Not all were complimentary about the organizational plans in place, for example one participant noted that the plans were ‘half-baked and confused’. It is important to note that there were no responses which indicated that any of the participants were driving the change or making key decisions for their organizational contexts relating to the impact of Brexit.

### Perspectives on the Brexit decision

**Fig 1:**
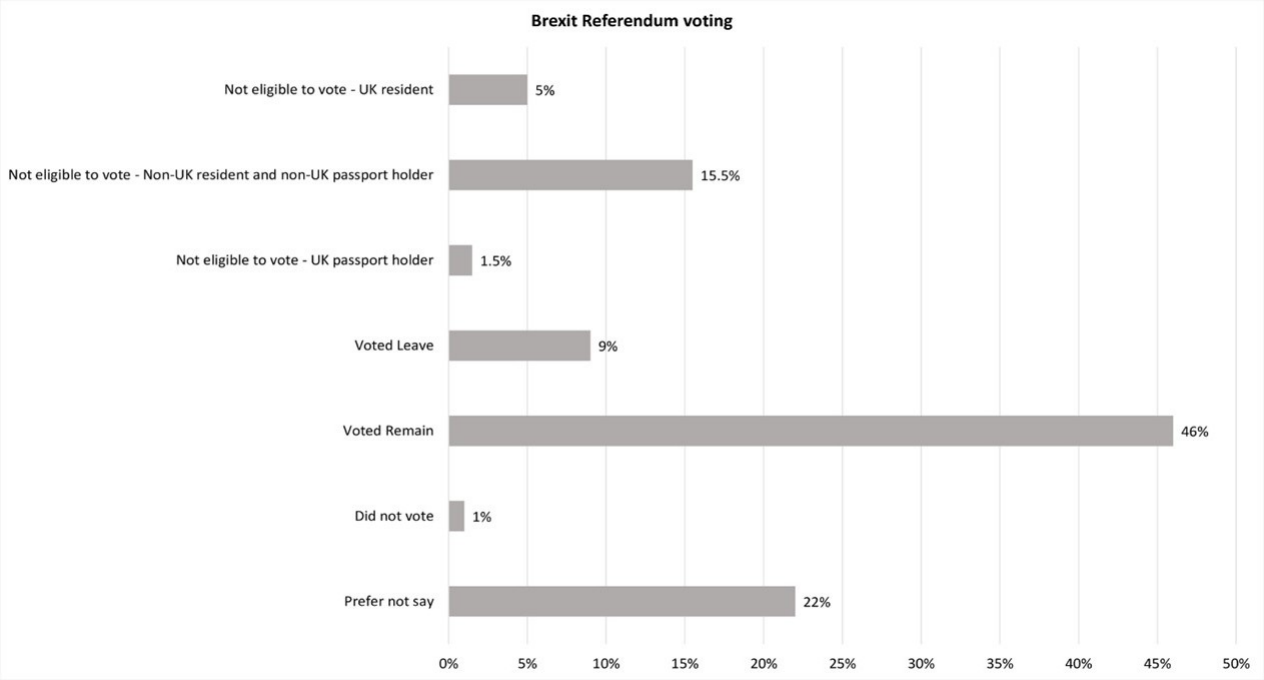
2016 Brexit referendum voting position as indicated by 2018 survey participants.

Of the 56% of participants eligible to vote or willing to state their position on Brexit, 46% indicated that they had voted to remain with only 9% indicating that they had voted to leave and 1% not voting (Fig 1).

**Fig 2:**
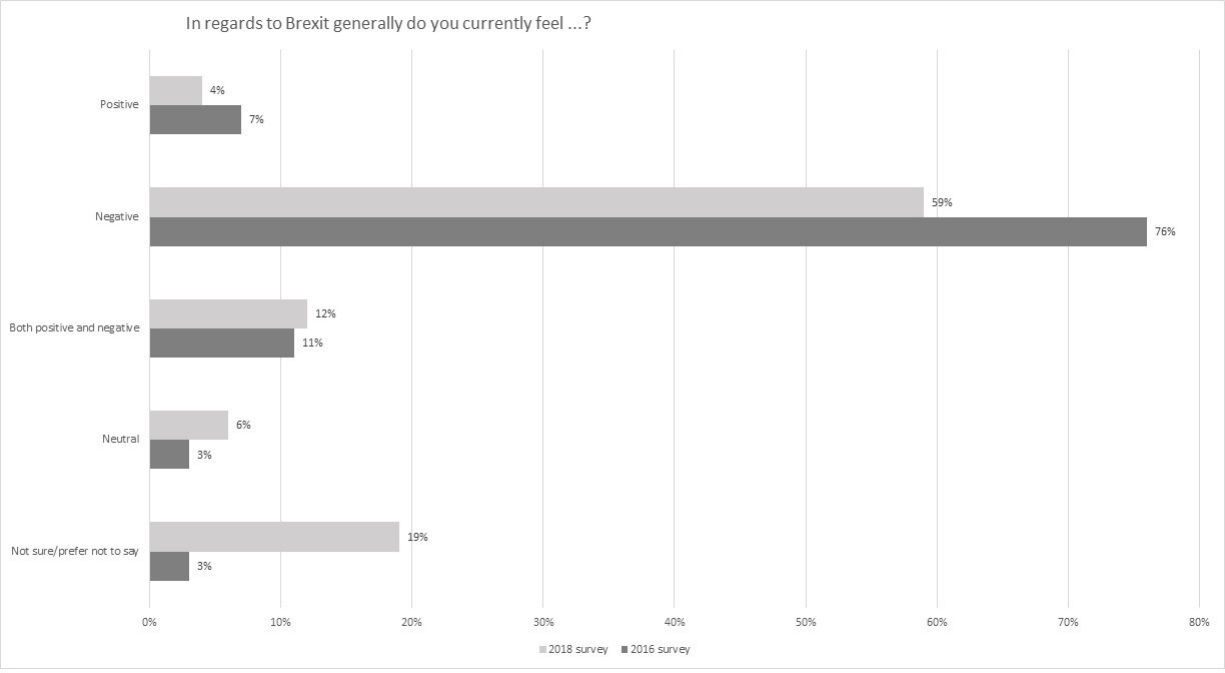
General perspectives on the Brexit decision comparing 2016 and 2018 survey results.

**Fig 3:**
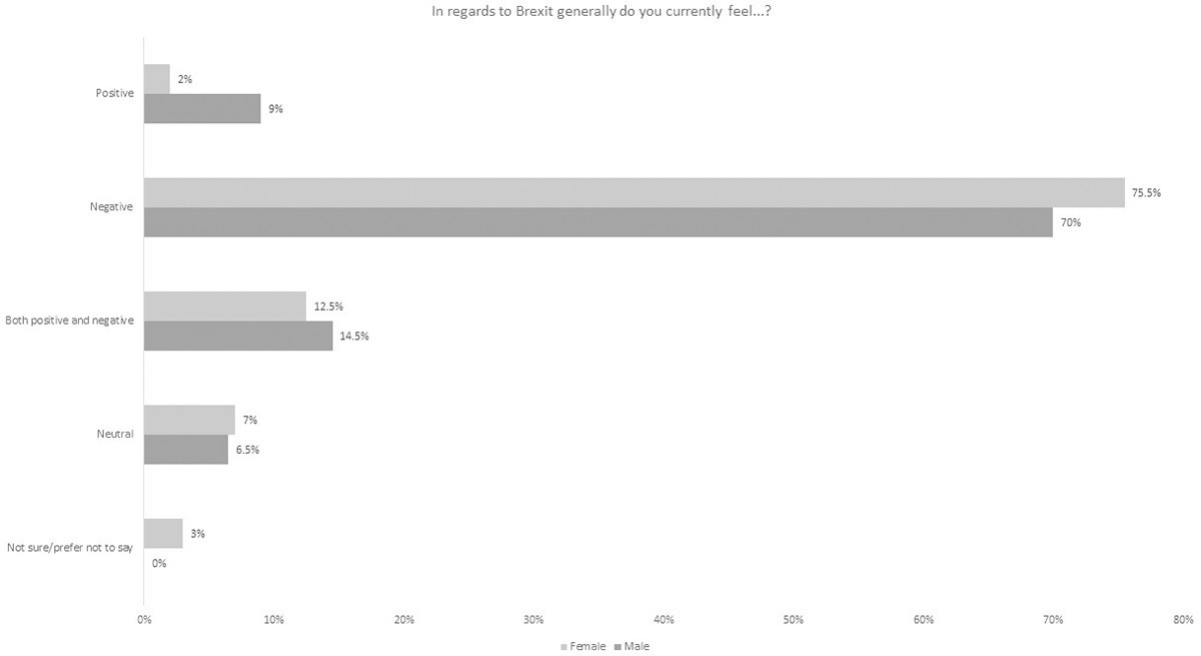
General perspectives on the Brexit decision by gender.

**Fig 4:**
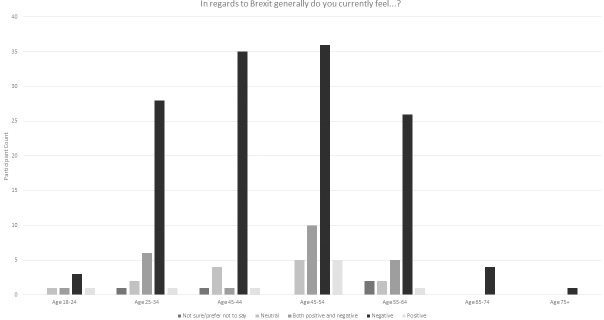
General perspectives on the Brexit decision by age.

59% of 2018 survey participants indicating a view saw the Brexit decision negatively (Fig 2). This was a decrease from the 2016 survey participants but largely due to a significant shift in those who were neutral, unsure or preferred not to say (25% as compared with 6% in 2016). Only 4% viewed the decision as positive, which was a drop of 3% from the first survey. Looking more generally at the impact of Brexit, the dominant perspective on this was that it was negative. The negative view was the modal value irrespective of gender or age group (Table 4, Figs 3 and 4).

**Table 4:** Breakdown of 2018 survey general perspectives on the Brexit decision by demographics by participant count (modal values shaded in grey).

	Male	Female	Age 18–24	Age 25–34	Age 45–44	Age 45–54	Age 55–64	Age 65–74	Age 75+
Not sure/prefer not to say	0	3	0	1	1	0	2	0	0
Neutral	5	7	1	2	4	5	2	0	0
Both positive and negative	11	12	1	6	1	10	5	0	0
Negative	54	73	3	28	35	36	26	4	1
Positive	7	2	1	1	1	5	1	0	0

The following sample comments evidence the wide-ranging views on the process:

“it's a disaster!”

“Where is the still distraught and very negative option?”

“I have complex sentiments”

“I believe there will be immediate losses but long term gains.”

“Presents an opportunity for people to democratically control the destiny of the country”

“Powerful undemocratic forces in the UK are colluding with the EU to undermine Brexit and try to keep the UK shackled to the EU. If these forces can be defied I shall be very positive about leaving the EU”

“This survey seems to assume that Brexit is a done deal but it isn't. I'm actively involved in the campaign for a referendum on the final deal which, if successful, will provide voters with the opportunity to mandate the Government to withdraw Article 50.”

**Fig 5:**
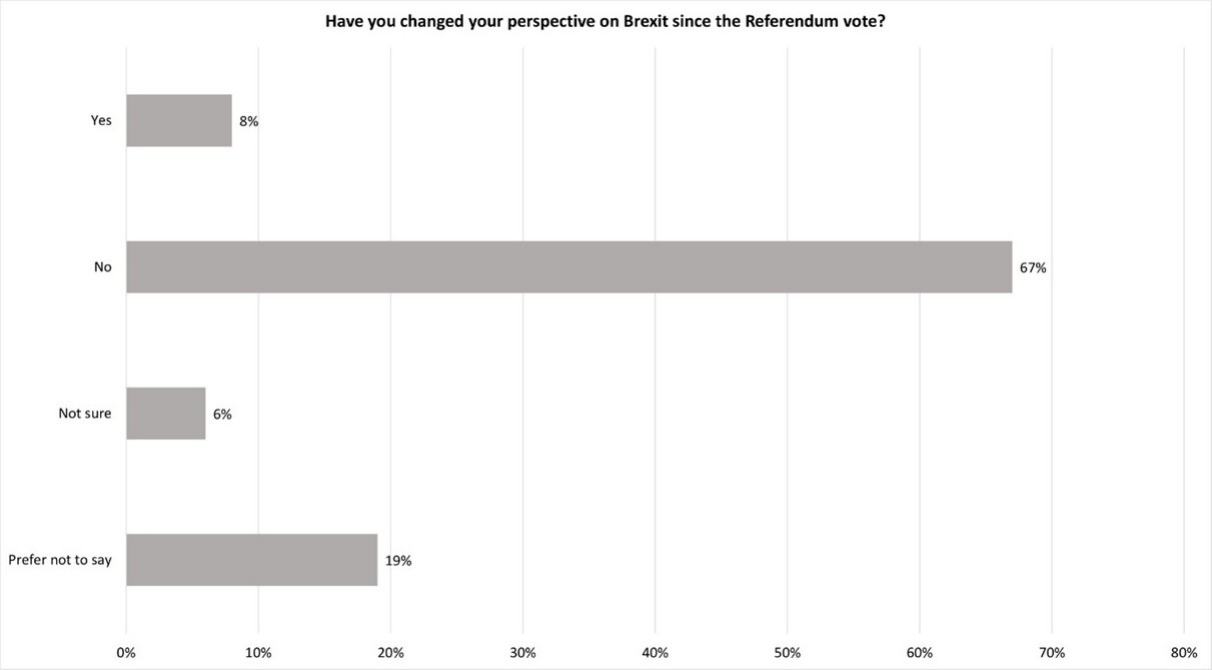
Changed perspectives on Brexit.

Related to this question, participants were asked if they had changed their perspectives on Brexit since the Referendum debate (Fig 5). 67% had not changed their original viewpoint. A significant number of participants (19%) did not want to comment or were now unsure about their views. 8% of participants had changed their view.

Many comments indicated anger at those involved in the process from the UK and EU. Examples of quotes which indicated changing, conflicted and angry perspectives include:

“Voted remain to keep status quo, based on limited EU knowledge. Since referendum, I have learnt so much more about what we will lose and how the EU actually works in detail that I am now even more pro-remain.”

“become reconciled to it happening and see there are opportunities arising from change (even unwanted change)”

“Although much more tired by it all. It is never ending divisive and uncertain. I have heard vast sums are being expended in Government on this—it is such a waste of money. When people were told about the sums sent to EU no one quoted cost of all this change. It is difficult to see how any of this can turn out ok”

“The Leave faction fought the referendum on a false prospectus and nobody explained the difficulties and complexities of untangling the UK from Europe. The Electorate did not understand what it was voting for and now one year on we still don't know where we are going or where we will end up. I am even more strongly opposed to Brexit than I was before.”

“Let's be honest, the negotiations really are going badly. And it isn't even a surprise! The issue of the Irish border was raised during the referendum—and yet people claim in the news etc that they weren't told.”

“My position has moved from being mildly pro-'Remain' to mildly pro-'Leave', even as I can see the threats and risks that lie ahead. I never have been sure what to think of the whole thing! One thing I certainly know, though, is that I dislike the souring of the tone of the national conversation. Both sides have been to blame in this regard, and I'm afraid I have personally been put off the 'Remain' camp by its bitterness since the vote.”

“I think the EU has not helped the UK turn back. If we have another vote I think more people will vote leave both to respect the initial democratic process and due to the EU tone.”

**Fig 6:**
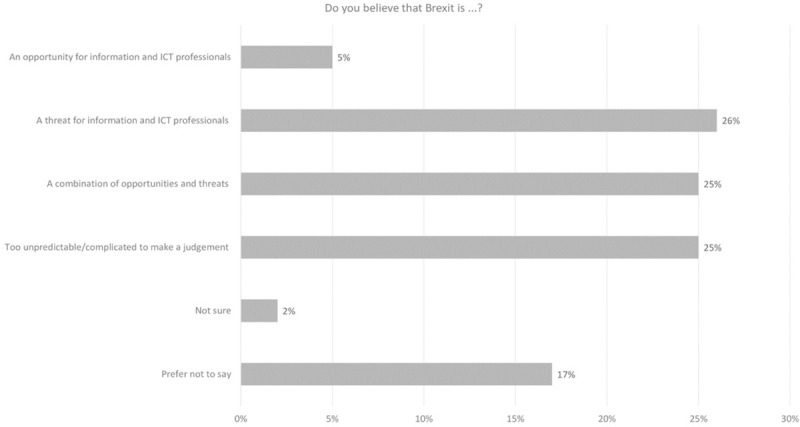
2018 survey perspectives on Brexit from an ICT professional perspective.

**Fig 7:**
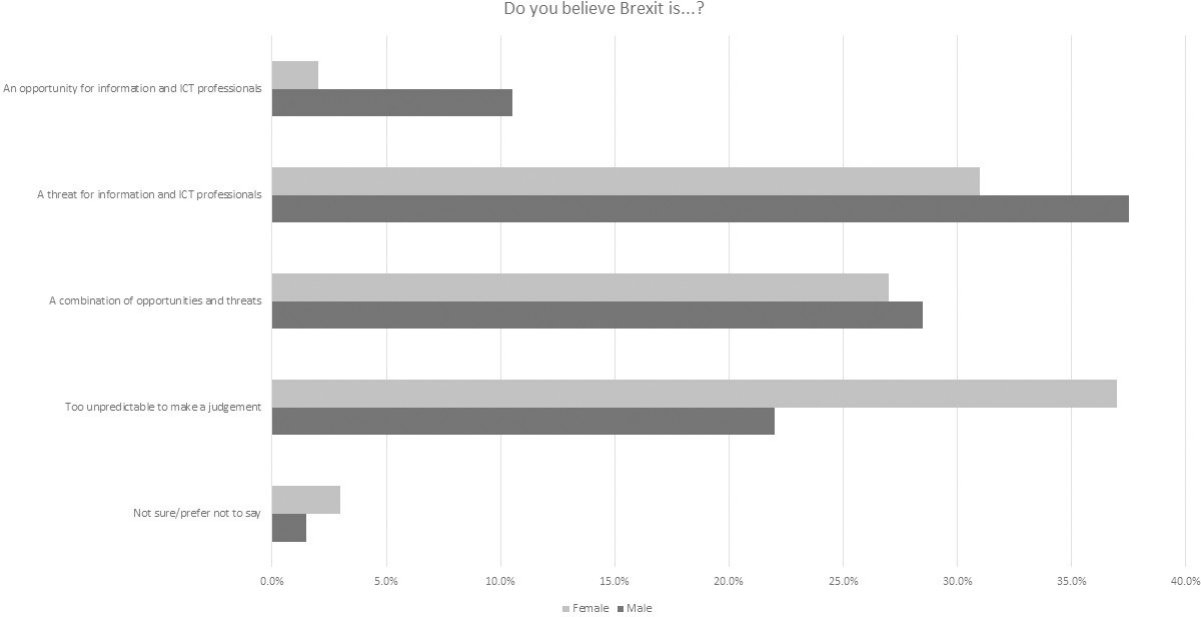
Breakdown of 2018 survey perspectives on Brexit from an ICT professional perspective by gender.

**Fig 8:**
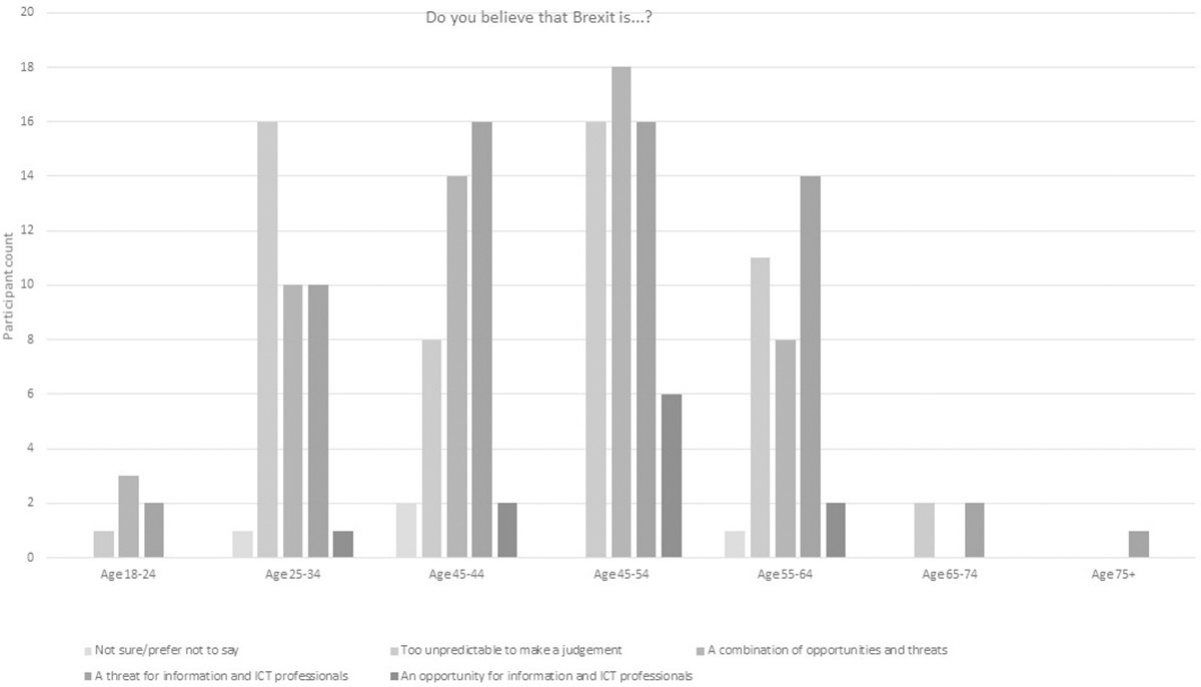
Breakdown of 2018 survey perspectives on Brexit from an ICT professional perspective by age.

In regard to participants’ personal perspectives on the impact of the Brexit decision for ICT, 5% saw it as an opportunity with 26% seeing it as a threat (Fig 6). Many participants were either unsure (2%), preferred not to say (17%) or ackowledged the complexities of the potential outcomes for ICT with 25% seeing it as too unpredictable to judge and 25% seeing it as a combination of threats and opportunities. When broken down by gender, the modal value revealed that female participants perceived the change to be too uncertain to judge for ICT professionals in terms of opportunities. The highest modal value for male counterparts perceived it as threat which did have a high participant score for female participants (Table 5, Fig 7). There were some different perspectives on this from across the age spectrum (Fig 8).

**Table 5:** Breakdown of 2018 survey perspectives on Brexit from an ICT professional perspective by demographics (modal values shaded in grey).

	Male	Female	Age 18–24	Age 25–34	Age 35–44	Age 45–54	Age 55–64	Age 65–74	Age 75+
**Not sure/prefer not to say**	1	3	0	1	2	0	1	0	0
**Too unpredictable to make a judgement**	17	36	1	16	8	16	11	2	0
**A combination of opportunities and threats**	22	26	3	10	14	18	8	0	0
**A threat for information and ICT professionals**	29	30	2	10	16	16	14	2	1
**An opportunity for information and ICT professionals**	8	2	0	1	2	6	2	0	0

**Fig 9:**
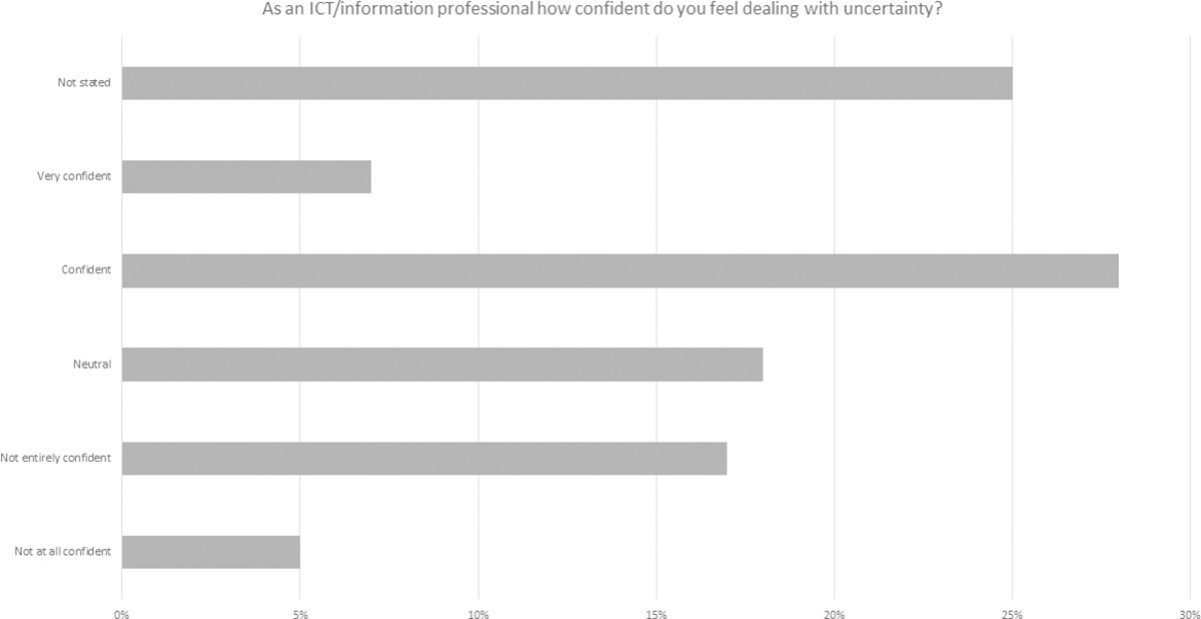
ICT professional confidence in dealing with uncertainty.

**Fig 10:**
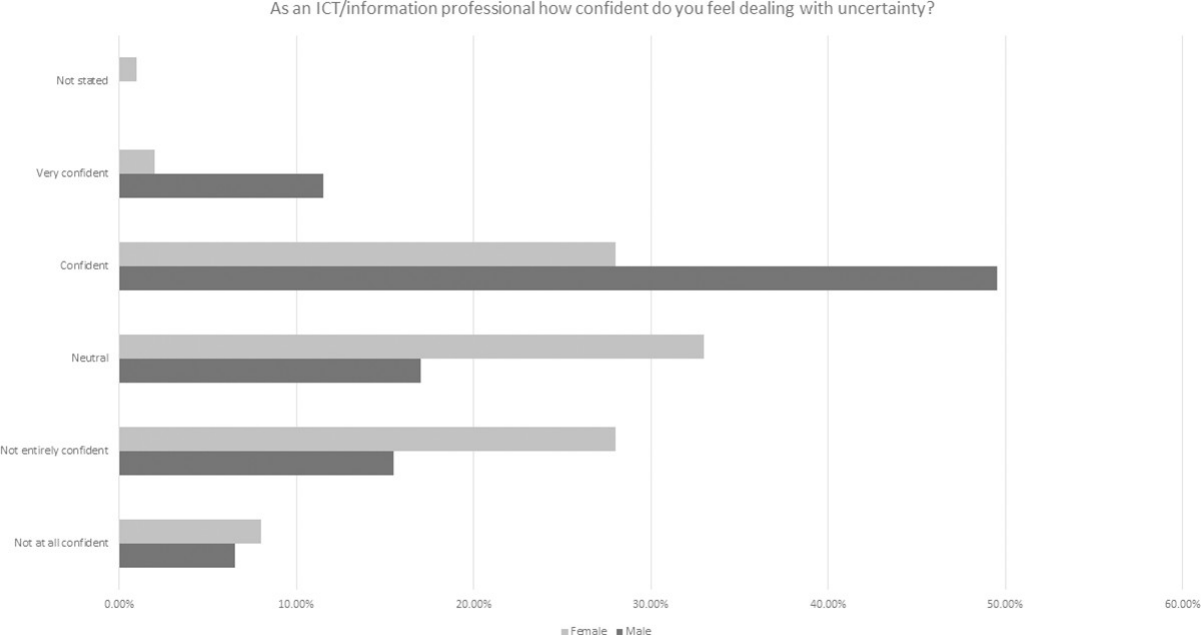
ICT professional confidence in dealing with uncertainty by gender.

**Fig 11:**
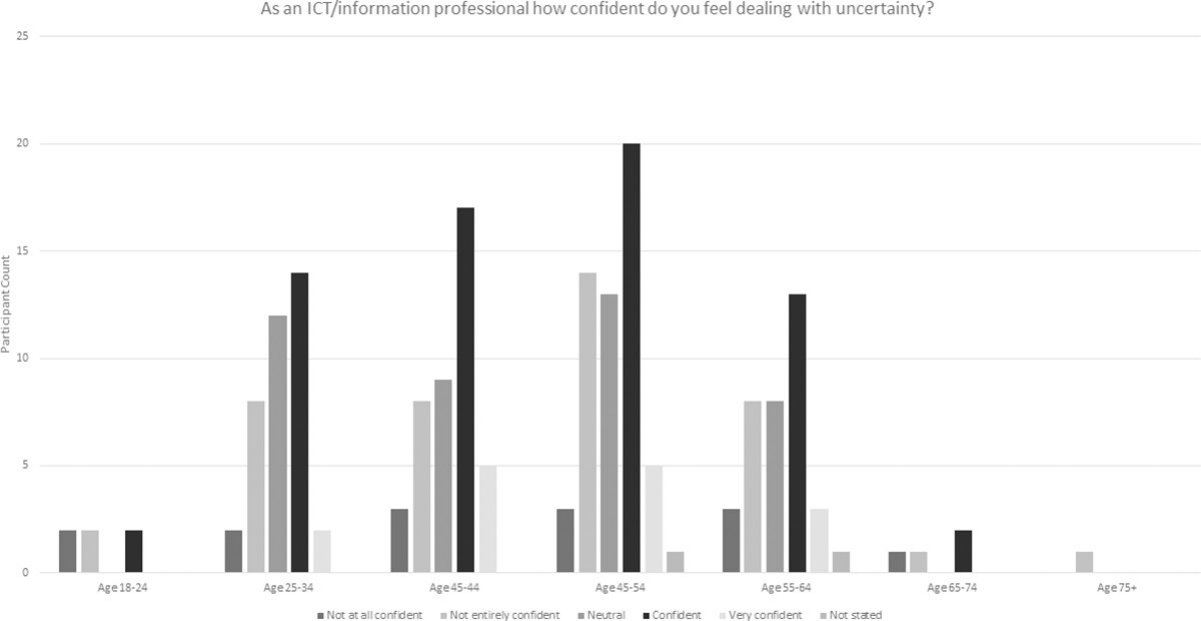
ICT professional confidence in dealing with uncertainty by age.

An additional question was asked in this survey in order to better understand ICT professionals’ attitudes to change and uncertainty (Fig 7). 28% of participants indicated that they were confident, with 7% very confident. However, 25% of participants did not state an answer. When the responses were broken down by gender and age there were some distinctions (Figs 9, 10 and 11 and Table 6).

**Table 6:** Breakdown of 2018 survey perspectives on ICT professional confidence in dealing with uncertainty (modal values are shaded in grey).

	Male	Female	Age 18–24	Age 25–34	Age 35–44	Age 45–54	Age 55–64	Age 65–74	Age 75+
**Not at all confident**	5	8	2	2	3	3	3	1	0
**Not entirely confident**	12	27	2	8	8	14	8	1	1
**Neutral**	13	32	0	12	9	13	8	0	0
**Confident**	38	27	2	14	17	20	13	2	0
**Very confident**	9	2	0	2	5	5	3	0	0
**Not stated**	0	1	0	0	0	1	1	0	0

A number of the comments indicated that uncertainty and change differ and that in the case of Brexit it was very difficult to gain any control on aspects of the change:

“Entirely depends on the nature of the uncertainty as there are always things outside of one's immediate control. The concern I have primarily with Brexit is that its complexity and interconnected nature to virtually every aspect of society means that no one at all is in control.”

“general uncertainty and dealing with change, is part and parcel of being an information professional and technology. However, Brexit terms are a political uncertainty which shifts this answer to not entirely confident. In particular over the land border being virtual or soft, only technology will address that and the implications are not being fully addressed by government negotiating with the EU.”

“Change and uncertainty are not the same. I can deal with change and the narrow parameters of managed uncertainty but this is a different kind of uncertainty”

“I can handle change but feel uncertainty is problematic as this potentially implies someone else/some force is entirely in control. However I can deliver risk management frameworks.”

These comments also reflected a theme that was repeated in response to other questions that the politicians, in the UK Government in particular, were not providing a clear narrative and framework or advice to respond to the change.

### Opportunities and threats

Open codes were created from the survey analysis of the responses to the open questions (S1 Table). 227 open codes were linked to opportunities, whilst 163 open codes were linked to threats. There was some overlap in the themes identified. Table 7 provides an overview of the themes with the highest coded counts. It was significant to note that participants were able to list a number of opportunities even when being negative overall about the process. Nine participants did specifically record that there were no opportunities and 27 participants indicated that reversing the Brexit decision was the only opportunity. These comments are not included in Table 7. It lists the top 30 most frequently occurring codes which represents all codes with a count of 8 or more. The coding frequency ratio for opportunities compared to threats was 1.27:1 which was a positive shift from the 1:2.02 ratio in the 2016 survey. It was interesting that, despite the uncertainty, the participants were able to consider and advocate for potential opportunities. This is potentially explained by Appelbaum et al’s analysis of change that even when people are negative about change, they can still focus on finding positive solutions, particularly if the potential consequences of unsuccessful change are very significant. However, this was a qualitative survey and the ratios are not conclusive about perspectives. In addition, it is possible that those who felt they could not see opportunities did not choose to complete the survey.

**Table 7:** Highest frequency occurring coded themes (opportunities and threats) from the survey linked to STEEPLE factors.

***OPPORTUNITIES***									
		**STEEPLE FACTOR**							
**NO**	**CODE**	**S**	**T**	**E**	**E**	**P**	**L**	**E**	**TOTAL**
1	Global perspectives and collaboration	25	33	21	18	20	15	14	146
2	Certainty—a clear and certain plan and agreement soon	6	7	6	4	6	6	4	39
3	Maintenance of rights for non-UK workers including academics	7	7	7	5	5	0	5	36
4	GDPR compliance and development	5	8	3	0	3	8	4	31
= 5	Artificial Intelligence (AI) and key technological advancements including Fintech, blockchain and infrastructure	2	10	6	1	1	4	4	28
= 5	Constructive dialogue and debate	4	4	4	4	4	4	4	28
= 5	UK freedoms and control	4	7	5	0	3	9	0	28
8	International standards	3	5	3	4	3	3	3	24
9	Workable borders for ICT sectors	2	7	3	2	3	3	2	22
10	UK connection and strengthening Commonwealth networks, trade and structures	6	5	3	2	4	0	0	20
11	Retain connections between UK and EU	3	2	2	4	3	3	2	19
= 12	Needs for ICT solutions, services and products	2	14	0	0	1	0	0	17
= 12	UK control and direction	2	4	3	2	2	2	2	17
= 14	Capacity for UK to develop new laws and regulations	2	3	2	0	1	6	2	16
= 14	ICT and information literacy education	4	6	3	1	1	0	1	16
= 16	UK control and direction of research funds	4	4	3	1	1	0	1	14
= 16	Limit/lose information rights law	5	2	4	0	0	0	3	14
18	New ICT skills	5	5	3	0	0	0	0	13
= 19	Links to China	3	3	3	0	2	1	0	12
= 19	UK promotion of democracy	5	0	2	0	3	0	2	12
21	Better ICT understanding and recognition	2	5	2	0	1	1	0	11
= 22	Efficiency driven and supported by ICT	1	4	3	2	0	0	0	10
= 22	Open Government and Open Data	2	2	1	1	2	1	1	10
= 24	Fluctuating pound providing new opportunities, e.g. for investments and exports	2	3	3	0	1	0	0	9
= 24	Innovation stimulated by change and uncertainty	0	4	0	0	5	0	0	9
= 24	New UK tax models	0	3	4	0	0	0	2	9
= 27	ICT skills work visas	2	4	2	0	0	0	0	8
= 27	Ethical ICT frameworks	1	2	1	1	1	1	1	8
= 27	New labor markets	3	3	2	0	0	0	0	8
= 27	New laws	2	2	0	0	0	4	0	8
***THREATS***									
		**STEEPLE FACTOR**							
**NO**	**CODE**	**S**	**T**	**E**	**E**	**P**	**L**	**E**	**TOTAL**
1	Isolation/xenophobia	11	24	19	13	14	12	13	106
2	GDPR loss	9	15	14	4	9	14	4	69
3	Uncertainty and lack of direction and control	8	6	10	8	18	4	4	58
4	Loss of skills, talent, specialisms and as a result innovation	15	15	15	0	0	0	0	45
5	Loss of labor pool	13	13	13	0	4	0	0	43
6	Loss skilled jobs/work from UK	8	10	13	3	0	0	0	34
7	Less research/loss EU research funding	6	10	7	2	2	2	2	31
8	Loss of freedom of movement	7	7	7	2	2	2	2	29
9	Lack UK Government action	4	4	4	4	4	4	4	28
10	Lack of mature and constructive debate	5	5	4	3	3	3	3	26
11	Borders	4	5	6	3	2	2	2	24
12	Loss of collaborative opportunities	6	6	6	0	3	0	0	21
= 13	Polarized politics	4	4	3	2	4	1	2	20
= 13	UK profile/power diminished	3	3	3	3	3	3	2	20
= 15	Loss of ethical laws and transparency	2	4	0	3	2	3	5	19
= 15	UK loss of EU networks	4	4	4	1	4	1	1	19
17	UK move to USA policies and reliance	2	3	3	2	2	2	2	16
= 18	Loss of students to UK	5	5	5	0	0	0	0	15
= 18	Loss of trade for EU and UK	3	3	3	0	3	3	0	15
= 20	Corporate entities leave UK	2	2	2	2	2	2	2	14
= 20	Lack of UK Government Expertise	2	2	2	2	2	2	2	14
= 20	More Trumps	2	2	2	2	2	2	2	14
= 23	Time	2	2	2	2	2	2	1	13
= 23	UK unattractive to (skilled) workers	4	5	4	0	0	0	0	13
= 25	Environmental impacts	0	2	2	4	0	0	3	11
= 25	Technological developments including AI and drones	2	2	2	1	1	1	2	11
27	Misinformation	3	1	1	1	3	0	1	10
28	UK loss of service work	3	3	3	0	0	0	0	9
= 29	Complexity laws	2	2	2	0	0	2	0	8
= 29	Cyber security threat/hacking/warfare	2	2	2	0	2	0	0	8
= 29	IPR problems for UK	2	2	2	0	2	0	0	8
= 29	Loss of EU funding for a range of industries	2	2	2	0	0	2	0	8

The themes developed from the appreciative inquiry workshops focused on identifying opportunities for action. They had a synergy with those comments coded through the survey (Table 8).

**Table 8:** Themes identified from the appreciative inquiry workshop linked to STEEPLE factors.

STEEPLE FACTORS	THEMES
**Social-cultural**	• Growing ICT skills in schools through education linked to practice. • Workforce frameworks to attract the best ICT skills and talent to the UK. • Planning for future societal needs and linked ICT requirements/skills.
**Technological**	• Pushing new knowledge and ICT development, e.g. the UK can lead in AI, blockchain and Fintech. • Research and development (R&D) and innovation frameworks and funding. • UK leading on ethical data storage, management and deletion. • Collaboration in areas which require international strength, e.g. cyber security.
**Economic**	• UK infrastructure to support ICT delivery. • New international business models and tax frameworks, e.g. UK committee to consider business models and UK R&D tax reliefs and state aid to targeted sectors.
**Environmental**	• Smart living underpinned by ICT driven global environmental solutions. • Sustainable and managed data storage and infrastructure.
**Political**	• A Brexit plan and an ICT Brexit roadmap to reduce uncertainty and enable planning. • Active and evidenced Government engagement with the ICT domain. • Supporting international study in the UK.
**Legal**	• Optimizing legislation and regulatory frameworks for an ICT driven world. • UK leading on highlighted international standards, e.g. ISO/TC 307 work on blockchain.
**Ethical**	• Laws which preempt potential ethical considerations aligned to the pace of ICT development. • Ethical ICT delivered from across ICT practice and into society, e.g. ICT sector whistleblowing on bad data practices.

### Certainty, polarization and mistrust

A number of issues emerged from reflection on the themes and coded comments. An immediate concern was a desire for certainty and decisions regardless of Brexit perspectives. This was highlighted as a clear need for a range of reasons including business planning, economic stability, labor force planning and wider social cohesion. In terms of action and expertise the UK Government was particularly criticized. Comments around these issues included:

“Again I am not sure the Government are properly linking up all the pieces in the puzzle.”

“Lack of Government expertise and action.”

“We know there are Government discussions occuring but please get a move on and produce more substantive action before it is all too late.”

“It is not possible to deal with a lack of facts. This makes planning very hard. So whilst I can change in response to situations the current waivering is not great”

In terms of delivering that change a constructive dialogue was seen as required to move on the agenda positively. However, it was noted that diplomacy had been lacking on all sides and, in addition, the media were not assisting the debates:

“It would be really helpful if the media were more responsible and messaged people more appropriately rather than causing divisions. The media's entertainment track has undermined serious collaboration and discussions.”

“In terms of threats and opportunities when sitting on your own it is far easier to list out lots of problems of which I see many—the world is a scary place at present—but to build a better world we need better discussions and projects delivered across all communities. It feels like negativity sells news—the media have a role in developing more positive rather than alarmist dialogues.”

The development of polarized politics and mistrust of official information was perceived to be a significant issue. Moves to agendas on the left and right were both highlighted as potentially dangerous with a growing concern about the possibility of “more Trumps”:

“The politics has been shaken up and there is a move to right and left wing extremes. Whilst there are problems in the middle there everything is quite scary.

### Isolation and xenophobia

Isolation and xenophobia were highlighted as key threats across the EU and for the UK more particularly. There were fears that isolationism would create growing hostile environments and, in addition, underpin an environment which could foster dangers around cyber security and warfare:

“Loss of [UK] opportunities for partnerships across Europe; loss of transferable status; isolationism; broken relationships, huge increase in unemployment, rising xenophobia.”

STEEPLE—The whole cyber crime scene and warfare through this is all really worrying. The Web and our ICT world has been positive and optimistic but we may need ICT borders to keep us safe. Will our bank accounts be hacked, our energy supplies cut down, our hospitals stopped? Will we move back to physical management and paper to keep us safe?”

### Global collaboration

In the longer term the greatest opportunities were seen to align to the promotion and delivery of global collaboration to develop internationalized networks, trade, labor and talent exchange. A particular example was that, “environmental change relies on global agreement—the EU made the Kyoto agreement happen.” It was observed that whilst clearly the EU is a larger political entity than the UK, it had nevertheless been seen to be protectionist, which has at times limited innovation and global development. In addition, the structures of the EU were noted to make progress and change slow, and decision making at the lowest common denominator level to gain agreement. The ability for a defined, agile and unique UK set of law, policy and investment planning provided critical UK opportunities. However, more significantly in this context, was the perceived need for the UK to have allies including maintaining EU relationships and strengthening ties with other countries such as China, the USA and Commonwealth nations. In the ICT context the need for global collaboration, law and international standards was particularly identified as the following comments illustrate:

“An opportunity: collaboration with non-European countries, perhaps English-speaking Commonwealth countries, which might (from a governmental perspective) reward investment and good relations. There is a great deal of youth and energy in these places, and strong cultures. Two countries that come to mind are India and Nigeria. Much fruit might be yielded for both partners in seeing what each can offer the other.”

“We need to have better more serious global debates.”

“EU legislation is restrictive and [an] overreaching. Without having to adhere to the different laws opportunities will be widened. When we sell digital subscription to EU customers we have to add VAT which makes the service less attractive. We do not have to do this with customers in America, Australia and other countries that are outside the EU. This will create a more attractive level playing field. We live in a global world so a focus on all markets and not just working within the EU is a positive outcome. Brexit is great for politics as it means that our government can't use the EU as an excuse to hide behind. All laws and decisions will be based on our national interests and will be made in the UK. Brexit was about claiming back control of our own laws in order to enhance democracy.”

There were mixed perspectives on the benefits of the current form of protecting personal data as set out in the EU’s General Data Protection Regulation [57], although a majority of participants viewed the EU legislative system as socially responsible and wanted to ensure the continued protection of rights and values. Others saw opportunities to lower or alter some compliance areas and to reduce worker rights to make the UK a more attractive prospect for business to locate. New compliance and regulation were seen as providing leverage for new networks and relationships globally. ICT was noted to be globally delivered and rely on complex international legal contexts. However, politicians were deemed to be lacking in skills and knowledge to properly understand ICT needs including laws/regulation, policies and education required to support and effectively deliver this change. An example was provided in one of the workshops of the failure of the USA Congress to properly cross examine Mark Zuckerberg at his Congress appearance in 2018. The need to better engage with ICT experts and to be open about this process was deemed critical. Ironically, whilst noting the need for global collaboration most comments continually reflected on personalized and national contexts.

### Role of ICT

At the workshops, the ICT sector was seen as somewhat of a false premise as ICT encompasses a number of functions ranging from IT support within particular private and public sector contexts through to ICT driven industries producing software, hardware, content and services, including broadcasting, cyber-security, e-commerce, gaming, Fintech and telecommunications. As ICT is an integral part of all sectors, it was suggested that it is perhaps best described as a knowledge ‘domain’.

Events triggered by Brexit across the UK and EU27 were identified as requiring ICT support to deliver solutions. For example:

“Technology and research are needed for making this massive change.”

“We need information professionals to deal with, support and deliver on the Referendum and all the cascade of impacts”

Within the workshops the need for new systems around supply chains and border controls were identified as areas where ICT could provide solutions. In addition, the need for information literacy skills and ethics was highlighted as having been evidenced to aid better citizen engagement for political change:

“information literacy instruction, which should now be leaving the academy and the workplace to be taught as a basic universal skill.”

“A great opportunity to raise the profile of information literacy (S) and information ethics (E)”

The development and pace of technological change was noted as a much bigger concern for society than Brexit. For one participant “a bigger threat than any BREXIT or remain is AI” whilst for others AI represented a significant pathway for ICT and societal development:

“We have a strong ICT sector including robotics, AI and machine learning. We need to lead with clear frameworks so we can develop these further.”

For a number of participants, the UK was seen as being well positioned to capitalize on ICT development in a range of contexts, including AI, blockchain and Fintech. To do this the UK needed to be able to continue to attract ICT global labor and talent. This was noted to require political recognition of ICT skills for visa purposes. Supporting student visas and exchange was seen to be part of the process of educating and developing a strong skilled workforce. At a developmental level ICT education in schools was seen as needing to be re-thought to ensure that the workplace needs were better met. In this context the model of classroom teacher-led learning was seen as requiring review. It was noted at one of the workshops that school-based teachers cannot keep up with the pace of change in this subject area and industry exchange needs to be fostered and funded.

In regard to developing skills and moving ICT forward an opportunity was seen to be the continued EU27 and UK research collaborations which are mutually beneficial and, in particular, remain possible as the EU research position includes others beyond the EU context. In addition, it was stated that the UK could re-envisage its research and innovation funding focuses if outside the EU. This could include revising competition law, R&D business/tax breaks, VAT and other tax laws. Business models were seen to have fundamentally shifted in the last 20 years with technology changing political and social control. New business frameworks and models were felt to be needed. It was suggested that the UK could become an ethical leader in this regard. In the latter context ethical and environmental agendas, including data capture, ownership, storage, maintenance and deletion, were seen to be a global concern. However, ICT was noted by some participants as not borderless and supply changes, together with inflation, were real concerns for UK based businesses. The following interwoven comments reflect these complex issues:

“S = English is still a dominant language and will remain the key mode of communications in IT and information resources after Brexit. T = UK has some of the best games industry specialists in the world and will be unaffected by Brexit. May be able to get tax breaks after leaving EU. L = things like GDPR and freedom of information could grow. IPR will stay important and may need further investment in new regulatory environment.”

“The opportunities are really highlighted through focusing on analysing each component of the business model to work out likely areas of increased profit and losses. Some parts of the ICT business costs are more flexible and it is possible to try to take advantage of the weaker pound. This may make our work more competitive when bidding against other suppliers. As an existing business one works through things …”

### Action to support the ICT domain

The emergent themes from the workshops and survey when analyzed together can be distilled into a series of high-level opportunities for the ICT domain, viz.:

1. UK/EU Brexit plan and an ICT Brexit roadmap to reduce uncertainty and enable planning across both the UK and the EU
2. Active and evidenced Government engagement with the ICT domain
3. UK infrastructure to support ICT delivery
4. Pushing new knowledge and ICT development
5. UK workforce frameworks to attract the best ICT talent
6. Growing ICT skills in schools
7. Planning for future societal needs and linked ICT requirements/skills
8. Supporting international study in the UK
9. R&D and innovation frameworks and funding
10. New international business models and tax frameworks
11. Optimizing legislation and regulatory frameworks for an ICT driven world
12. UK leading on international standards
13. Smart living underpinned by ICT driven global environmental solutions
14. Ethical data storage, management and deletion
15. Ethical ICT delivered from across ICT practice and into society

For each one corresponding concerns around the threats posed by uncertainty, division, ICT ignorance, misinformation and generally lack of engagement with a wide range of ICT threats including cyber warfare were evident in the survey. They form an agenda for further exploration and action by policy makers and are the subject of the briefing document that makes the case for the UK Government to work with a range of ICT experts to support and grow the ICT domain through the changes Brexit presents [58].

### ICT change in the Brexit context

Reflecting on the change literature a number of issues are highlighted around the problems for the Brexit change and this is reflected in the perspectives of participants’ comments. The conditions for Applebaum et al’s commitment to change exist [18] and the participants’ responses did reflect the three different levels of engagement with change (affective, normative and continuance commitment). However, what change Brexit requires is uncertain and this is reflected in the comments, both explicitly and in terms of the lack of contextual detailed information and leadership at a national and organizational level. The crafted positive messages, information and strategic plans for action are lacking. The high-level information required to manage change, such as the planned basis of trade relationships between the UK and EU, is still unknown. This has created uncertainty. Furthermore, the change may not happen with calls for a further Referendum–the People’s Vote. As such people have not been transitioned forwards emotionally with appropriate information. Many people are still in the stages of grief, loss and anger which are part of the stages of change within a number of the change management models [13, 14, 15]. If, ultimately, the goal for effective change is to move people towards informed optimism, as highlighted by Lawrence [17], then the conditions for this do not yet exist. This is reflected in the data gathered in the study. However, the potential for opportunities nevertheless exists within these uncertainties and this was highlighted in responses, even though it is not possible to fully engage with these more proactively given the lack of information on what the change means, as highlighted by Rumsfeld’s statement about the problem of the “unknown unknowns” [38].

## Conclusions

In regard to perspectives on the Brexit Referendum, whilst a high percentage of the participant responses were not personally positive about the decision, there had been a significant shift from the first survey to a recognition of the complexities of analyzing the situation. The analysis revealed that, whilst the participants saw the benefits and opportunities in global collaboration, most viewpoints were presented from a national perspective. This was further evidenced by the larger numbers engaging with Brexit from UK bases or with UK nationality and the limited engagement from non-UK contexts. In terms of the survey coding and workshop themes, the high-level emergent ICT opportunities from the workshops did mirror those comments coded from the survey.

In regard to understanding the change triggered by Brexit for ICT, even at this mid-point in the process participants were still very uncertain on what the Brexit change would mean. This is reflected in the sample of ICT professionals’ personal and professional perspectives on the change triggered by Brexit. As noted by participants this uncertainty presented a different context for responding than dealing with a more clearly established future position, which could enable planning and engagement with change. The responses indicated the ability of those participating to contribute constructively and positively in spite of their wider viewpoints which they still acknowledged. The participants were able to consider and advocate for potential opportunities with a ratio of opportunities to threats of 1.27:1. This is potentially explained by Appelbaum et al’s analysis of change that even when people are negative about change, they can still focus on finding positive solutions, particularly if the potential consequences of unsuccessful change are very significant, as in this case. Many opportunities and threats were not necessarily dependent upon the Brexit change but rather existed in spite of Brexit. The conditions of the Brexit process have yet to reach a stage where there is the potential to foster positive change through information, clear messaging and facts. The survey is part of a longer-term study. Using the same methodological approach, further data will be collected assuming the UK leaves the EU. As the facts of the Brexit process emerge more clearly, and the many ‘unknown unknowns’ are replaced by more ‘known knowns’ and ‘known unknowns’ [32], it will be possible to see whether certainty influences perspectives on the change in what is a complex transition. As such any data gathered after Brexit will be able to gather more explicit data on ICT professionals’ personal and professional perspectives on the change triggered by Brexit in terms of opportunities and threats.

## Supporting information

S1 Fig (PDF)

S2 Fig (PDF)

S3 Fig (PDF)

S1 Table Coding 2018. (PDF)

S2 Table (XLS)

S3 Table (XLS)

S4 Table (XLS)

S5 Table Sample of responses. (PDF)
