# Supplementary material for: Engaging with change: Information and communication technology professionals’ perspectives on change at the mid-point in the UK/EU Brexit process
Source: PLoS One. 2020 Jan 6;15(1):e0227089. doi: 10.1371/journal.pone.0227089 (PMC6944360; doi:10.1371/journal.pone.0227089)
Supplement: S1 Fig — (PDF) [file pone.0227089.s001.pdf]

Research Overview

Background: Brexit presents a significant change for the UK and the EU. This shift in national relationships has potentially global ramifications. However, those professionals who work in the Information Communication and Technology (ICT) sector and/or manage information, whether in the public or private sector, are well practised at harnessing opportunities in response to fast changing environments.

Research purpose: This survey aims to identify information professionals' perspectives on opportunities and threats for information management/ICT in the light of Brexit. The work will inform a better understanding of information management/ICT responses to large scale and complex change. The survey is open to anyone. Although Brexit was triggered by a UK Referendum, we are keen to hear information professionals' perspectives from around the globe on the impact of this change taken from differing contexts. We hope people will contribute in a constructive manner even when noting concerns.

This survey follows an initial survey launched on the first working day after the Brexit Referendum. The results are freely available in the Journal PloS One . We aim to publish the results of this survey quickly in an open access journal and in addition produce a policy briefing for the information/ICT sector. Additional papers and newspieces may be produced. We plan to repeat the study to gather further data as the picture evolves.

Data collection: There are 21 questions (all optional). The survey will take 10-20 minutes to complete depending on how much you wish to write. We know that you are busy and appreciate your time. You can exit the survey and your previous comments will be saved so that you can go back and complete it when you have time. The survey will close on 27 April 2018.

Every effort will be made to anonymise contributions. We are not collecting IP addresses. The information you provide will be retained securely and anonymously and will be kept in line with UCL research data management and retention policies. The findings will be written up in the form of an article, including sample quotes. The full data set will not be made available in order to enable free and frank comments to be provided.

Delivery: The research is a partnership between the iSchools at UCL (Dr Elizabeth Lomas) and Northumbria University (Professor Julie McLeod).

The survey is being facilitated by Dr Elizabeth Lomas e.lomas@ucl.ac.uk

Thank you for your help.

Brexit context

1. Did you previously complete the first Brexit survey we released...

- Yes
- No
- Not sure

2. In regards to voting in the Brexit Referendum which is applicable ...

- Not eligible to vote - UK resident but not eligible to vote
- Not eligible to vote - non-UK resident and non-UK passport holder
- Not eligible to vote - non-UK resident but UK passport holder
- Voted 'Leave'
- Voted 'Remain'
- Spoiled ballot paper
- Did not vote
- Prefer not to say
- Comment:

3. In regards to Brexit generally, do you currently feel...?

- Positive
- Negative
- Both positive and negative
- Neutral
- Not sure

Comment:

4. Do you believe that Brexit is...?

- An opportunity for information and ICT professionals
- A threat for information and ICT professionals
- A combination of opportunities and threats
- Too unpredictable/complicated to make a judgement
- Not sure

Comment:

5. Have you changed your perspectives on Brexit since the announcement of the original Referendum vote in 2016?

- Yes
- No
- Not sure
- Prefer not to say

Please add any comments regarding how you have changed your views and in particular how your views have shifted in terms of how this impacts ICT/information professionals:

Demographics

6. In which Brexit voting region or country outside the UK do you reside?

Comment:

7. Which is the country that most closely defines your nationality?

Comment:

8. In which age range are you?

- 18 to 24
- 25 to 34
- 35 to 44
- 45 to 54
- 55 to 64
- 65 to 74
- 75 or older
- Prefer not to say

9. How do you prefer to identify your gender?

- Female
- Male
- Transgender
- Prefer not to say

Please add any comment you wish to make:

10. What is your current occupational status?

- [ ] Employee
- [ ] Self-employed
- [ ] Unemployed
- [ ] Student
- [ ] Volunteer
- [ ] Retired
- [ ] Other (please specify)

11. Which best describes your profession/ job function/ area of study/former profession if retired?

- [ ] Academic
- [ ] Administrator
- [ ] Archivist
- [ ] Business analyst
- [ ] Cyber security
- [ ] Data manager
- [ ] Information manager
- [ ] IT support
- [ ] Information security expert
- [ ] Lawyer
- [ ] Librarian
- [ ] Management consultant
- [ ] Marketing manager
- [ ] Mergers and Acquisitions expert
- [ ] Records manager
- [ ] Software developer
- [ ] Web designer
- [ ] Managing Director
- [ ] Manager
- [ ] Consultant
- [ ] Project manager
- [ ] Other (please specify together with your job title)

What is your job title?

____

12. Approximately how many employees are there within your organisation?

- ○ 0-9
- ○ 10-49
- ○ 50-250
- ○ 250-1,000
- ○ 1,000-10,000
- ○ 10,000+

13. Do you manage any staff?

- Yes
- No

14. If you are working or volunteering is your organisation a global entity operating from sites spread across the World?

- Yes
- No

15. Does your organisation have any plans in the light of Brexit?

- Yes
- No
- Don't know
- Are you able to briefly outline what these are?

16. As an ICT/information professional how confident do you feel dealing with uncertainty?

Not at all confident	Not entirely confident	Neutral	Confident	Very confident
○	○	○	○	○

Please add any comments you wish to make:

Brexit ICT perspectives

Opportunities and Threats
We are seeking to gather your current thoughts on OPPORTUNITIES and THREATS for information and ICT professionals in the context of the the Brexit decision. The 'STEEPLE' model factors (listed below) provide some prompts in terms of particular factors which you may wish to consider.
Social-cultural factors (S)

Technological factors (T)

Economic factors (E)

Environmental factors (E)

Political factors (P)

Legal factors (L)

Ethical factors (E)

17. Can you identify any STEEPLE opportunities for information/ICT professionals in the light of Brexit? [Leave fields blank where you do not have any ideas]

18. How could these opportunities be harnessed? [Leave fields blank where you do not have any ideas]

19. Can you identify any STEEPLE threats for information/ICT professionals in the light of Brexit? [Leave fields blank where you do not have any ideas]

20. How could these threats be minimised? [Leave fields blank where you do not have any ideas]

21. Do you have any other comments to make regarding information/ICT change and/or the impact of Brexit more generally?
