# Supplementary material for: Engaging with change: Information and communication technology professionals’ perspectives on change at the mid-point in the UK/EU Brexit process
Source: PLoS One. 2020 Jan 6;15(1):e0227089. doi: 10.1371/journal.pone.0227089 (PMC6944360; doi:10.1371/journal.pone.0227089)
Supplement: S2 Fig — (PDF) [file pone.0227089.s002.pdf]

Engaging with change: ICT professionals' perspectives on change and opportunities in the light of Brexit

Project Information Sheet

Research Partners: UCL (University College London) and Northumbria University

Details of Study:
This research aims to explore the opportunities and responses to change from the information/ Information Communication and Technology (ICT) sector in dealing with the far-reaching challenge triggered by the UK's Brexit decision. Those professionals who work in the ICT sector and/or manage information, whether in the public or private sector, are well practised at harnessing opportunities in response to fast changing environments. Whilst Brexit is a UK decision this shift in national relationships has potentially global ramifications and therefore this study is hoping to draw in perspectives from the UK, EU and all nations.

This research is being conducted using surveys and workshops. The surveys will be delivered over c.3 years with Appreciative Inquiry workshops being held in Newcastle and London. It is in regards to the workshops that we are hoping to involve you.

Appreciative inquiry is a strengths-based approach, intended to explore and develop the positive perspectives of participants through an exploration of their social world in an inclusive way, obtaining data about their perceptions and experiences. It seeks to establish a basis for positive development and change. The framework for focusing the participants will be a STEEPLE model. STEEPLE is an acronym for seven factors—Socio-cultural, Technological, Economic, Environmental, Political, Legal and Ethical. This model is a common management tool used in commercial circles, including ICT settings. We will be mapping information/ICT opportunities connected to these factors.

Research outcomes
The outputs from this work will be an open access journal article. In addition, it is intended that there will be a policy briefing paper, additional presentations and short news pieces for the information/ICT industry. The work should identify opportunities for the ICT sector in the light of the Brexit challenge and extend the evidence base to inform policy makers.

How can I help?
We would like to invite you to participate in one of the Appreciative Inquiry workshops. You have been selected as someone who can contribute to developing a picture around the issues of change in the context of Brexit.

Will I be named or identified in the research?
You can choose whether you want to be named or remain anonymous. If you chose to be anonymous you won't be identified in any publications, publicity and presentations. You will be given a unique number which will be used instead of your name. Any contributions you make that could identify you because they are very personal or specific to you will not be used. If you name anyone else in your contributions, then we will make every effort to anonymise the data.

Will the workshops be recorded?
We will be recording selected parts of the event when you are feeding back. You will be made aware that this is happening. This is being done in order to include quotes to provide context for the findings in publications. The audio recordings will not be shared.

Will you take photographs?
We will not take any pictures of individuals. Photographs of the materials developed during the workshop may be shared publicly in research outputs.

How will we hold your information?
All personal information (e.g. your contact details) is collected and stored in accordance with the Data Protection Act 1998, the General Data Protection Regulation (from May 2018), UCL's Data Protection Policy and Research data management policy. UCL is the data controller and Elizabeth Lomas will oversee the data management. As a co-researcher, the data will be shared with Julie McLeod at Northumbria University. However, it will only be accessible to Lomas and McLeod.

The audio files and transcripts will be stored on a research database that only Elizabeth Lomas and Julie McLeod will be able to access. The data will be in line with UCL retention policies. All information will be held securely on UCL storage.

This research has been approved by the UCL Research Ethics Committee and is registered with the UCL Data Protection Officer. The UCL Data Protection Policy can be found at: http://www.ucl.ac.uk/legal-services/policies. The UCL Data Management Policy can be found at: http://www.ucl.ac.uk/library/research-support/research-data/policies.

Who will have access to my contact details?
Your contact details will be held securely in an encrypted password protected spreadsheet managed by Elizabeth Lomas and Julie McLeod. We will not pass your contact information on to anyone else without your permission.

Can I withdraw from the research?
Yes, you can withdraw from the research at any time without giving a reason.

What will happen to the research?
We will use the information we collect to produce an open access journal article which we will share with you. In addition, we will produce a sector policy briefing. We will speak about the research at meetings and conferences, and publish news pieces and articles about what we have found.

Are there any risks to being involved?
We will be open and honest with you throughout the research and do everything we can to minimize risk to you. You can ask us to remove parts of your contribution if you would prefer them to be confidential provided you inform us ahead of any publications.

What are the benefits of being involved?
The benefits of being involved are the value of considering the Brexit challenges and opportunities with others. Through engaging in a constructive dialogue with other leaders in the ICT sector preconceptions about the Brexit process can be challenged. In addition, it is hoped that the work will influence and contribute to the public facing evidence base regarding the implications and opportunities relating to Brexit. You may choose to be credited for taking part in this workshop or to be anonymised in any outputs from these discussions.

Project contacts:
University College London: Dr Elizabeth Lomas: e.lomas@ucl.ac.uk
Northumbria University: Prof. Julie McLeod: julie.mcleod@northumbria.ac.uk

If you have any comments or questions, please do not hesitate to get in touch.

Engaging with change: ICT professionals' perspectives on change and opportunities in the light of Brexit

Consent Form

By signing this consent form, you are agreeing to take part in the Engaging with Change project. A separate information sheet is attached which should be read in conjunction with this form.

Please tick each of the boxes to confirm you have understood the conditions of taking part. If you have any comments or questions, please get in touch with Elizabeth Lomas at e.lomas@ucl.ac.uk.

Confirmation of information received

I have read and understood the information provided in this consent form and the information sheet	
I have had an opportunity to ask questions and discuss the research	

Agreement to take part in the workshop

I agree to take part in the workshop	
You may keep and use my personal details to communicate with me about the research	

Recording of workshop discussions

I agree to the recording of the feedback sessions in the workshops. I understand I will be made aware during the workshop at which points recordings are happening.	

Use of contributions

I agree to the use of my contributions made in the course of this workshop being used in publications, presentations and other outputs. All quotations will be anonymized.	
I understand that photographs of the mappings produced during the workshop may be published	

Anonymity or acknowledgement (tick one box in this table)

I would like to be anonymous and referred to by a unique identifying code	
I would like to be credited by name as having participated in the research where appropriate	

Contextual information (please print any information you are happy to provide for context)

Please indicate the sector you work in. This may be used for context around your discussions, so please record this as you would wish it to appear.	
Please indicate your profession. This may be used for context around your discussions, so please record this as you would wish it to appear.	

Name (please print)	Email address and/or contact number
Signature	Date
