# Supplementary material for: Engaging with change: Information and communication technology professionals’ perspectives on change at the mid-point in the UK/EU Brexit process
Source: PLoS One. 2020 Jan 6;15(1):e0227089. doi: 10.1371/journal.pone.0227089 (PMC6944360; doi:10.1371/journal.pone.0227089)
Supplement: S3 Fig — (PDF) [file pone.0227089.s003.pdf]

Engaging with change: ICT professionals' perspectives on change and opportunities in the light of Brexit

Key Event Contact: Dr Elizabeth Lomas

Workshop Programme

11:30-15:00

This workshop involves a small number of participants who are experts from across a range of industry and academic contexts linked in to ICT. The participants include UK and overseas participants. The work is being conducted as an Appreciative Inquiry. This means that whilst we recognize there are concerns relating to the Brexit decisions, we will encourage the capture of opportunities that can influence a range of areas including for example policy, investment strategies and network development.

Outcomes of this work will include:

- Identification of opportunities for the ICT sector in the light of the Brexit challenge
- Development of ICT sector position(s) on the Brexit challenge
- Development of an evidence base of perspectives on opportunities to inform policy makers

It is intended to produce publications for a range of audiences including a policy briefing paper.

11.30	**Welcome, introductions and explanation of the workshop format** – Elizabeth Lomas & Julie McLeod & Elizabeth Lomas
11.45	**Activity: Mapping the opportunities**<br>Using a 'Steeple' framework opportunities will be mapped against the seven factors within the framework —Socio-cultural, Technological, Economic, Environmental, Political, Legal and Ethical. No prior knowledge is needed. [STEEPLE Factors are on the sheet below.]
12:45	**Feedback on the opportunities**<br>Discussion looking across the factors.
13:15	**Lunch**<br>Over lunch discussions will continue in order to further develop the picture.
14:00	**ICT highlights and conclusion**<br>To conclude key policy messages and actions will be identified.
15.00	*Workshop closes for participants.*

Thank you for taking the time to contribute to this event!

STEEPLE FACTORS*

Socio-cultural analysis

Socio-cultural factors (S) include customs, lifestyles, and values that characterize the society in which an organization/individual is operating. They can also include demographics of age distribution, population growth rates, level of education, distribution of wealth and social classes and living conditions. Socio-cultural factors may influence entrepreneurial spirit, fashions and consumer demands, the ability of a society to obtain resources or trade in certain ways due to consumer influence.

Technological analysis

Technological factors (T) refer to the rate of new inventions, development and changes in technology including software, hardware, networks and e-commerce. They can also include: methods of manufacture, distribution and logistics. These factors also include attitudes to research and research spending.

Economic analysis

Economic factors (E) represent the wider economy so may include: sector growth, levels of employment, consumer confidence, costs to the sector (e.g. hardware and licenses), interest rates and monetary policies, exchange rates, inflation, investment opportunities and research funding.

Environmental analysis

Environmental factors (E) include energy and resource-efficient goods (carbon neutral, recycling etc.) services and technologies and promotion of informed choices by customers. They also include threats from natural disasters.

Political analysis

Political factors (P) refer to the stability of the political environment and the approaches of political parties, sector interest groups and other stakeholders. This may include policies on tax, trade or the sector in general.

Legal analysis

Legal factors (L) include for example, domestic legislation, EU regulation, self-regulation, standard agreements, international trade agreements, national and international competition law, trade union agreements and consumer protection.

Ethical analysis

Ethical factors (E) include the moral expectations determining how individuals and organizations should operate. This is the domain of governance which extends beyond the strictures of law and influences approaches to accountability and confidentiality, bribery, intellectual property rights, brand and reputation.

*Provided with permission of Professor Alison Harcourt.
