# Supplementary material for: Engaging with change: Information and communication technology professionals’ perspectives on change at the mid-point in the UK/EU Brexit process
Source: PLoS One. 2020 Jan 6;15(1):e0227089. doi: 10.1371/journal.pone.0227089 (PMC6944360; doi:10.1371/journal.pone.0227089)
Supplement: S1 Table — (PDF) [file pone.0227089.s004.pdf]

CODING 2018

Oppurtunities axial codes/themes	Opportunities open codes
	[Remain/reversing Brexit]
Certainty - a clear and certain plan and agreement soon	Certainty
	Direction for business planning - economic stability - labour force planning
	Clarity soon for social cohesion
	Agreement of a clear plan
	Modelling routed forward
	Speeded up Government discussions
Positive change	Shake up - positive change
Responsible/Better informed Government personnel/politicians	Better Government Ministers - trained
	Move away from unbureaucratic elite
	Need for knowledgeable civil servants
	Responsible politicians
	Government ICT knowledge
Punish racism	Punish racism
Transparent politics	Transparent politics
Environmental benefits	Shrinking trade and helping the enviroment
New political agreements	New political agreements
Strong deals for all	Strong deals for all
Constructive dialogue and debate	Constructive dialogue
	Diplomatic language and behaviours

	Mature debate<br>Positive narratives
Agreements	Formal agreements
Transparent trusted news	Facts- jornalism- move media away from entertainment news to factual news/communicty news<br>More responsible media<br>Positive trusted channels for communication not Facebook Google etc<br>Transparency
Mediated help accessing information	Mediated help finding inforomation
Brexit changes needs	Data - facts - evidence<br>Data analytics<br>Researchers<br>Lawyers<br>Technologies and ICT processes
Funding	Budgets -Finance UK going to a wide range options not NHS
Minimise cuts	Rally against cuts
Customs union	Maintain export market - customs union for goods<br>UK stay in the customs union
EU UK collaboration	EU UK collaboration
Retain connections between UK and EU	Retain connections between UK and EU
Fluctuating pound providing new opportunities, e.g. for investments and exports	Weak pound for exports

	Economic fluctuations for investments
UK lose EU VAT	UK lose EU VAT
New economic models and financial structures	New economic models<br>New finance rules<br>New models of investment<br>Rethinking economics
New trade deals - agreements	New trade deals - agreements
New ways of thinking	New ways of thinking<br>Non-siloed western ideas
Meritocracy	Meritocracy
UK promotion of democracy	Democracy - UK - set example with Referendum
UK freedoms and control<br>[NB: research aggregated separately]	Free thinking UK<br>Freedom - UK - autonomy<br>Control - UK - legal and programmes<br>Control - UK research funds<br>Control - UK - marine resources<br>Lack of EU bureacracy in UK
Cooperation	Cooperation - industry
Coopetive funding	Cooperative funding arrangements for industry
Government information portal	Information portal from Government
New collaborations	New collaborations

New networks	New networks
UK connection and strengthened Commonwealth networks, trade and structures	Australia -markets<br>Australia - networks<br>Australia - Commonwealth<br>Commonwealth - markets<br>Commonwealth - networks<br>Commonwealth-political structures
[Note Asia link]	India - skilled labour<br>India - markets<br>India - networks<br>India - Commonwealth<br>Nigeria - networks<br>Nigeria - youth
Links to Asia	Asia-learning<br>Asia-markets - networks
Links to China	China-links<br>China - learning<br>China-skilled - labour<br>China-skilled - markets<br>China-skilled - networks
Mexico - skilled labour	Mexico - skilled labour
Russia	Russia-cooperation
Switzerland	Switzerland - models<br>Switzerland - networks

	Switzerland - economics
USA	USA - business links<br>USA - markets<br>USA - networks<br>USA - political links
Global perspectives and collaboration	Gobal academic links - shaping global perspectives - collaboration<br>Global best practice and knowledge<br>Global collaboration in industry<br>Global connection and collaboration - esp for environment<br>Global legal agreement<br>Global networking<br>Global professions<br>Global learning
UK learning form global networks	UK learning form global networks
Global skills	Global skills
Global movement	Allow global movement
Freedom of movement	Maintain freedom of movement UK-EU
Labor mobility	Labor mobility<br>Global skills mobilisation
New labor markets	New labor markets
Lose immigrant workers in UK	Loss immigrant workers - new UK job opportunities

Lose UK worker rights	Lose worker rights - make UK more competitive
Union power	Union power
ICT skills work visas	Skilled work visas - ICT skills recognition
Government amd ICT linked Action	Government 'Action' linked to ICT<br>Political/tech cooperation
Agility	Agility
Artificial Intelligence (AI) and key technological advancements including Fintech, blockchain and infrastructure	AI<br>AI ethical frameworks - agile<br>AI lawmaking<br>Blockchain<br>Fintech<br>Data Science<br>DNA ids<br>Robotics<br>Machine learning<br>Internet of Things (IoT)<br>Tracking software<br>ICT infrastructure advancements
Maintenance of rights for non-UK workers including academics	Maintain rights for non-UK/EU workers including academics
Alternative research models	Altenative research international models<br>Alternative research funding models structures
Maintain research funding	Maintenance research funding

UK control and direction of research funds	New UK research agendas<br>UK invest research money back esp data science, AI and centres of expertise<br>UK control and direction research funds
Strong academic and research community	Strong academic and research community<br>Promote research professionals
Lose students from UK visa statistics	UK lose students from visa stats
ICT and information literacy education	Information literacy - ICT education
Information professionals needed	Information professionals needed
Independent experts	Independent experts
New ICT skills	New ICT skills<br>Data analytics and date science skills
Growth technical skills	Grow technical expertise- not philosophers
New forms of ICT education	Education ICT skills focus - attainment<br>Educational shift
UK improvement	UK - improve at what we do/upskiling
Workable borders for ICT sectors	Border agreements - workable borders<br>Borders driving tech and law<br>Borders for security/cyber security<br>Seamlsess borders<br>Settled border questions

	Minmise global borders<br>Passporting and borders drive technology
Passporting	Passporting - incl banking commitments
ICT frameworks	Clearer farmeworks - business - law - ICT<br>Establish IT frameworks and policies
Deregulation	Deregulation
Digital Asset Management and law	Digital Asset Management (linked to international law)
Digital Brexit Champions	Digital Brexit champions - UK first in digital
Efficiency driven and supported by ICT	Efficiency driven and linked to ICT
New investment	New inestments in new types of ICT<br>New investments in regulatory frameworks
English	English as a language<br>English language IT
Ethical ICT frameworks	Ethical social media governance<br>Ethical ICT frameworks - storage - maintenance - ownership - deletion - capture<br>Informaiton ethics
Inclusive bodies	Inclusive bodies
Estonia ICT models	Estonia - ICT models
Virtual services	Virtual services

Better ICT understanding and recognition	Better ICT understanding and recognition
Support for ICT	ICT support
Measure and account for ICT	Measure and account for ICT
ICT growth as part of change	ICT growth in pushing change
Needs for ICT solutions, services and products	Tech for agriculture<br>Tech solutions - services - products needs in wake Brexit
Technology supporting society	Technology to support society, e.g crime prevention, drones etc
Technology supporting industry	Technology to support wider range of industries
Open Government and Open Data	Open data - open research - open Government
Oral history	Oral history
ICT efficiency	ICT linked to efficiency - UK - EU
International standards	International standards - panel memberships
Maintain EU standards	Maintain EU standards
Agreed EU - UK legal cooperation	Agreed EU -UK legal cooperation
Capacity for UK to develop new laws and regulations	Capacity for UK to develop new laws and regulations
UK GDPR adequacy	UK GDPR adequacy

Growing information Rights law	Legal - growing IPR<br>Legal - growing FOI<br>More international rights law - privacy - harmonisation<br>Property rights - DNA data<br><br>UK retention and development of information rights laws - GDPR-IP
Ggrowing information Rights law-GDPR compliance and development	GDPR development<br>GDPR -agreed data handling rules<br>GDPR compliance<br>GDPR growth
UK retention best EU laws	UK retain best EU laws - copyright<br>UK retention best EU social laws<br>UK - keep EU law
Limit/lose Information Rights law	Limit extension Information Rights laws<br>Lose FOI<br>Legal - Don't extend UK Information Rights Law<br>UK lose GDPR<br>Reduce surveillance laws
UK lose EU laws	UK lose EU laws<br>UK business advantes to loss some EU social laws
New laws	Legal review - change laws<br>Legal - competition law change<br>Legal - new laws -copyight-digital asset management
USA style laws	USA style laws

Less complex laws	Simplified laws
Transparent laws	Transparent laws as EU opaque
New regulations	New regulations
Patent agreements	Patent agreements
R&D/innovation push	R&D pushed<br>More innovation<br>R&D pushed for Creative industries<br>R&D
UK push competition frameworks	UK - aid competivie prices for UK services and products
Innovation stimulated by change and uncertainty	Innovation from uncertainty - change
Gaming industry	Gaming industry lessons/approaches
New UK tax models	New UK tax breaks<br>New UK tax changes<br>New UK tax models
Lose anti-comeptition laws	Lose anti-competition laws
Loyalty	Loyalty
New ICT alliances	New alliances fostered by ICT
SMEs pushed	SMEs pushed
Standardisation	Standardisation

Stop trade leaving UK	Stop trade moving out UK
Supply chain modelling	Supply chain modelling
Partnership projects	Supporting partnership projects
**Threats axial codes/themes**	**Threats open codes**
	Brexit fullstop
Uncertainty and lack of direction and control	Uncertainty<br>Lack of direction - control<br>Lack of EU/UK direction<br>Hysteria/panic<br>Lack knowledge EU/UK citizen status
Unrealistic public expectations	Unrealistic public expectations
Time	Time
Isolation/xenophobia	Isolationism - countries inwards facing<br>Xenophobia<br>USA turning in<br>Lack of languages<br>UK isolation from EU trading block
Fragmentation	Fragmentation<br>Lack of understanding across UK Remain/Leave and EU
Smaller networks	Smaller networks
Service complexities	Service complexities

Loss USA support of UK	Loss USA support for UK
UK move to USA policies and reliance	UK move to USA policies and reliance<br>Plundered UK
UK profile/power diminished	UK global profile downgraded - third nation<br>UK power diminished<br>UK locked out Europe influence
Gibraltar isolated and impacted	Gibraltar - suffers isolation - trade impacts
Disaster	Disaster scenarios
General disaster	General disaster
Race to the bottom	Lower standards - race to the bottom
Environmental impacts	Environmental impacts - issues not dealt with<br>ICT drain on earth<br>UK loss EU environmental requirements
Emnity EU/UK	Emnity between EU/UK - EU punishes UK as an example
International conflicts	International conflicts<br>Violence and unrest
Lack of mature and constructive debate	Lack of civilisized rhetoric - lack of mature debate<br>No debate<br>No positive narratives<br>Barriers to discussion and free speech

Misinformation	Misinformation<br>False information - fake news<br>Less reliance on evidence<br>Disputed information through conflicts
Loss transparency	Loss transparency
More Trumps	Influence President Trump<br>Move to USA Trump systems<br>More leaders like Trump
Polarized politics	Polarised politics<br>PM May<br>Move left<br>Move right - incl Trump
Borders	Borders<br>Irish border<br>ICT borders<br>Borders - trade barriers
Visa - creating barriers	Visas - creating barriers
Lack of efficiency	Duplicated efforts for EU/UK<br>Lack of efficiency
Bureaucracy	Bureaucracy
Lack of democracy	Democracy challenged
Lack of UK Government Expertise	Lack of Government officials expertise and action with proper skill sets and ICT need

Lack UK Government action	Lack of UK Government Action
Lack of action	Lack of action
Government failings	Government failings
Lack of government networks and links	Lack of Government networks-links
Loss of global networks	Loss of global networks
International travel cut	International travel cut
Less global communication	Less global communication
Cultural connections broken	Cultural connections broken
UK loss of EU networks	UK loss EU networks - collabration
Loss of control across EU	Loss of control across EU
Loss of collaborative opportunities	Loss of collaborative opportunities
Loss of freedom of movement	Loss of freedom of movement
Loss of freedom of services	Loss of freedom of services
Loss of freedom of speech	Loss of freedom of speech
Loss of UK and EU values	Loss of GB cultural value and EU value
Loss of choices in UK	Loss of choices in UK

Restricted markets	More restricted markets
Loss of trade for EU and UK	Loss of trade - EU - UK
Collapsed businesses	Collapsed businesses - incl craft businesses
Corporate entities leave UK	Companies move out UK - for EU - for other countries<br>EU taking business from UK
Joint working under threat	Joint working under threat
Loss of industry growth	Loss of industry growth - global and EU-UK
Loss of EU funding for a range of industries	UK loss of EU funding in regions
UK loss of service work	UK loss of service work
UK less competitive	UK less competitive<br>UK fining own organisations not others
Competition to UK	French national promotion<br>German national promotion<br>USA national promotion
Recession	Recession
Loss of economic security	Loss economic secuirty
Weak pound and currency fluctuations with rising interest rates	Weak pound<br>Currency flucations<br>Rise in interest rates

UK unable to afford imports	UK unable to afford imports
Cuts and austerity	Austerity<br>Cuts<br>Funding shortages
Lawyers	Lawyers
Legal costs for change	Legal costs for change
Resources and focus diverted by Brexit	Resources and attention wasted on Brexit
Inappropriate cuts	Wrong cuts
Loss of lifestyle	Loss of lifestyle
Loss of funding	Loss of funding
Higher cost of living	Higher cost of living
Loss of jobs	Loss of jobs
Loss of workers rights and freedoms	Loss of workers rights<br>Loss of worker freedoms
Loss of labor pool	Loss of labour pool
Loss of skills, talent, specialisms and as a result innovation	Loss of skills/specialisms - loss of innovation<br>Loss of talent and innovation
Loss skilled jobs/work from UK	Loss skilled jobs/work from UK

UK unattractive to (skilled) workers	UK unattractive to skilled workers - EU workers
Loss of professional networks and sharing	Loss of professional networks and information sharing
Slow down intellectual progress	Slow down intellectual progress
Less conferences in UK	Less industry and research conferences in UK
Diminishing respect for academics	Diminishing respect for academics<br>Strikes impacting academic reputation
Anti-education perspectives	Society moves to anti-education perspectives
Less research/loss EU research funding	Loss of research<br>Loss of EU research funding<br>Less collaboration so less research
Loss of students to UK	Loss of students to UK
Loss of R&D and innovation	Loss of innovation<br>Loss of R&D
Technological developments including AI and drones	AI<br>Banking tech<br>Blockchain - drain on resources<br>Drones
ICT slow down	Technological stagnation/slow down
ICT exclusion	ICT exclusion<br>Impact on services

UK lack of EU ICT trading networks and partners	UK lack of EU ICT trading networks<br>UK lack of EU ICT trading partners
Tech costs for UK on own	Tech expense for one nation
ICT professionals drawn into politics/biased	ICT professionals drawn into politics<br>ICT professionals biased by policies
Cyber security threat/hacking/warfare	Cyber security impacted<br>Breakdown security networks<br>State sponsored espionage
Data archives impacted	Data archives impacted<br>Data capacity problems for UK
Data Storage threats for personal data	DP/EU server issues for UK - convergence issues
Data barriers	Data barriers
Loss of roaming benefits	Roaming benefits
Complexity laws	Complexity law<br>Complexity privacy following separation
Complexity information management	Complexity RM regimes
Growing information rights law	Legal - growing GDPR<br>Legal - growing FOI<br>Legal -growing information rights laws<br>Legal - growing IPR

Loss EU laws	Loss EU laws incl patent law/freedoms and info access
Deregulation/loss legislation/restrictions	Deregulation/loss legislation<br>Lack of banking regulation impact ICT aspects<br>Loss restrictions/regulations
IPR promblems for UK	IPR problems for UK
GDPR loss	Legal - loss GDPR/data protection<br>Lack GDPR adequacy
	Loss GDPR - loss of data centres from UK to continental EU due to GDPR<br>Loss of personal data movement for UK
Loss of ethical laws and transparency	Loss ethical laws<br>Loss of transparency
Supply chain breakdown	Supply chain breakdown
