# Supplementary material for: Engaging with change: Information and communication technology professionals’ perspectives on change at the mid-point in the UK/EU Brexit process
Source: PLoS One. 2020 Jan 6;15(1):e0227089. doi: 10.1371/journal.pone.0227089 (PMC6944360; doi:10.1371/journal.pone.0227089)
Supplement: S5 Table — (PDF) [file pone.0227089.s008.pdf]

S5 Table Sample of responses

1.0	SAMPLE QUOTES - STEEPLE OPPORTUNITIES
1.1	Quite often EU legislation is complex, excessive, inconsistent, even extravagant, since to make good decisions when you have to cater for 28 different quarrelsome partners frequently proves to be a pipe dream. Brexit might possibly lead to streamlined processes and rules.
1.2	Honestly, I cannot see any opportunity
1.3	Clearly opportunities will abound almost by definition of the nature of Brexit. It's actually harder to think of areas to which this doesn't apply in the Brexit context. UK will attract brighter, more motivated & more innovative IT professionals post Brexit (P + S). Longer term economical/environmental factors will crystallise as UK's wealth of marine resources become more apparent. Legal factors will be significant in short/medium term. A new discipline, legal programmatic modelling and dispute resolution, will evolve (e.g. laws and treaties designed using computer modelling and court cases decided by AI). Personal property rights to extend to include SM data and ownership of DNA. DNA as a means of identification within 15 years. These latter 3 not strictly Brexit effects but developments in these areas catalysed by Brexit.
1.4	S = English is still a dominant language and will remain the key mode of communications in IT and information resources after Brexit. T = UK has some of the best games industry specialists in the world and will be unaffected by Brexit. May be able to get tax breaks after leaving EU. L = things like GDPR and freedom of information could grow. IPR will stay important and may need further investment in new regulatory environment
1.5	The opportunities are really highlighted through focusing on analysing each component of the business model to work out likely areas of increased profit and losses. Some parts of the ICT business costs are more flexible and it is possible to try to take advantage of the weaker pound. This may make our work more competitive when bidding against other suppliers. As an existing business one works through things. There are real issues in terms of understanding the supply chain for new businesses. The Ernst and Young report shows many banks are committed. This is one of my markets and a lot of UK wealth does flow from financial services so I do think that we need to be clear on the London passporting for the banking sector. It is an opportunity that we will keep GDPR as it means we should have adequacy and minimise some data impacts. We have a strong ICT sector including robotics, AI and machine learning. We need to lead with clear frameworks so we can develop these further.
1.6	T - technology and research are needed for making this massive change. The lawyers will do well.
1.7	T - Opportunity for UK to confidently focus on advancing IM and IT technology ourselves instead of relying on other nations. eg to foster data science and analytics centres of expertise
1.8	A great opportunity to raise the profile of information literacy (S) and information ethics (E)
1.9	If the EU collapses then the UK will have done well to get out. Ireland and Greece have suffered in the EU. It is not equal Germany and Spain dominate. The opportunities are global thinking
1.10	The opportunities for the UK are to be considerate to other countries and to build better partnerships. The UK needs to better understand and acknowledge the impact it had on certain countries when it went into the EU. At this time ICT was not part of the

	picture. With ICT if new alliances are forged ICT can help drive these and lessen the distance between new partners. The UK is relatively a small population but does have an educated workforce with some specialisms. There is a lot to be offered back to the UK from other countries. For those of us outside the EU we would want global stability. The EU has been inward facing. I would hope that both the UK and EU rethink some connections and concerns. As the globe does shrink we need to have better international mechanisms for discussion aside from just the global powershouses. It will be important for like minded countries to collaborate. This is why the Commonwealth has mattered because it has been about shared values. The concern is splintering and inward facing agendas at a time when certain nations are seeking to leverage greater power
2.0	SAMPLE QUOTES – HARNESSING STEEPLE OPPORTUNITIES
2.1	The issue with the Government reports and the witness statements (e.g. for DCMS) is that they do deal in the facts which are known. So for example we know that we are currently reliant on certain EU copyright laws/harmonisation. We know therefore that we need to consider the impact of a shift in UK status. These things are quite easy to plot and consider. What is not being proactively discussed in Government reporting in the ICT sector is the opportunities and areas for innovation. These are the areas of uncertainty which I perceive this survey to be about. In order to do this we need better sector by sector supply chain modelling. In addition we need thoughts on how the competition laws will change. Where can there be pushes to innovation? I have read the Bakhshi/Lomas work on Creative Industries - not sure if this two bits of work are linked? I would think that we do need to push R&D for this sector and really provide assistance in areas where the UK can take a market share. It is worth noting that the UK hit back at Canada in order to support the gaming industry. It would be good to see the figures on the success of this initiative. In terms of linking globally this happens in some domains. I think we need to think more about this.
2.2	There may be opportuntities to build links outside Europe that have not previously been harnessed, although that would depend on a number of both internal and external factors. As information professionals we are very good at creating and maintaining professional links, but I am not sure we use those to benefit our organisations. It seems to me that cross cultural and economic links are created by academics and businesses rather than by information workers. Citizens, researchers and stakeholders in the UK will have an even greater need to understand the nature of relations between the UK and the the EU after the country leaves. The much greater complexity of the relations will need an even greater need for mediated help to find and understand information.
2.3	The UK Government needs to be more agile to deal with the above. We haven't even got the DP UK law yet despite GDPR's implementation in May so this does not bode well for their ability to really move. I do think the Government needs to be far more proactive and clear in directions. It is the uncertainty that is very problematic. ICT professionals can deal with uncertainty but you must have some points of certainty to develop the intervening climb. The above make points about what we can harness. We can reach out and recruit skilled labourers. Promote our researchers
2.4	UK needs to build on and extend initiative in legal AI and DNA ids. Should also be strong part of new ISO standards in IT arena. Marine drone technology should be developed for fisheries, anti smuggling, environmental research/monitoring. New NI border solution should be designed carefully with an eye to marketing it to EU and beyond.
2.5	Set up trade deals, minimise borders, enable research, build constructive positive dialogues.

2.6	The DCMS produced a briefing paper but this is not as strong as some independent work in other sectors. It would help as a sector to have more independent reports for our sector so I hope this work will develop something. I would like clearer portals of information as there is information but it is very spread out. I note this work is also appealing to information managers and ICT professionals - lobby government for one clear portal of information from inside and outside Government. I would have liked to be able to draw in this survey to demonstrate some more complex ideas! Otherwise see above
2.7	By implementing Brexit fully and not being tied down to any restrictions that the EU may wish to impose on the UK
2.8	Look at the laws that might help. Look at new partnerships
2.9	They could develop mechanisms of laboral mobility with some countries that are developing high capabilities in this field for example (India, China, Mexico). The opening of relationships with other countries outside of EU, could help in increase knowledge and best practices in the field of cultural and educational cooperation.
2.10	Reverse the decision
3.0	SAMPLE QUOTES – STEEPLE THREATS
3.1	S - I feel library and information conferences and workshops held in the UK will become less attended by EU librarians as visas will be needed for international travel. This would be detrimental as diversity is important when networking. it is important to connect with people from other cultures and share information across all borders.
3.2	1. That understandable disappointment on the 'Remain' side might prevent a proper and sympathetic understanding - and recording - of the 'Leave' side, particularly heavily 'Leave'-voting regions, communities or organisations. My impression is that the information profession is broadly pro-'Remain', and as an archivist I am worried that the understandably strong feelings of 'Remain'-sympathising professionals (which, to some extent, I share) might affect their objectivity, or lead them into an overly political position. In particular they might be unreasonably suspicious of self-evidently British or English culture. 2. That 'Leave' voters will mistrust the information profession, if the fissure opened up by the referendum aligns itself with the level of formal education, as it seems to have done. 3. That an imprudent British government, free to abolish, or cease to enforce regulations that aid our work, exercises that freedom.
3.3	The danger is inaction and bureaucracy!! The threat is lack of global partners. The HEFE sector striking is not good - give the academics their pensions as we need to try to maintain our strong base as a leader in education and research.
3.4	It is not clear if the concept of GDPR adequacy will be sufficient for UK or if UK firms will need EU base to handle EU citizen data. In the hysteria of Brexit and Trump there are now barriers to discussion and free speech. This is very dangerous and we need to help manage this. The UK has a culture of punishing and fining its organisations in a way that does not exist in other countries including France, Germany and the USA. Ironically we do not fine businesses from these countries trading in the UK but we do penalise and shine a light on our own. We do need proper ethics and London has excelled at this. Cut us some slack and support us and if you penalise a UK firm then highlight the failings of others!
3.5	CLIFF CLIFF - CLIFF AHOY!!!!
3.6	In all areas it presents a threat. Leaving a large trading block comprising our nearest neighbours is economic idiocy. In socio-cultural terms Brexit comprises an empowering of xenophobia. In environmental terms it is clearly a bad idea to abandon the regulations that go with the EU in favour of the deregulation that goes with closer bonds with the US, as the hard Brexiteers argue (particularly at this

	moment, when the US environmental agency is shredding its own protections for the environment). As a citizen, this move to markets red in tooth and claw promised by the Brexit Tories is a threat in every aspect.
3.7	There is the obvious problems if freedom of services and movement go away, especially regards both filling open positions for companies and finding work for individuals. After Brexit, how will Data Protection and GDPR work with regards to where data/services are located. Will, for example, everything need moving from Irish/European datacentres, cloud regions, etc. to British ones? Is the capacity even available?
3.8	E = no idea, think it will be a disaster for 2-3 years and then level off. Growth unlikely in first 5 years. E = - no opportunities I can see P = who cares L = things like GDPR and freedom of information could grow. IPR will stay important and may need further investment in new regulatory environment E = ??
3.9	Economic - reduction in financial security and overall funds within the public sector Technological - less access to research Social cultural - less contact and easy involvement with our continental neighbours so many others its difficult to quantify encapsulated in my belief that there will generally be a lowering of standards, less reliance on evidence and greater reliance in flawed free-market thinking within all sectors
3.10	Workers Rights
4.0	SAMPLE QUOTES – MINIMIZING STEEPLE THREATS
4.1	Conciliatory and sober language in communications; in general a less politicised approach to our work.
4.2	Scrap Brexit and retain the status quo
4.3	If anyone had an idea of what the status for non-UK passport holders will look like and was able to communicate this information.
4.4	by supporting communications and projects beyond the Brexit through international or European organizations, institutions and associations by thinking that Brexit is not forever and the digital environment can easily maintain cooperation without taking into account the national barriers
4.5	At any level (national, local, private, third-sector, professional), UK bodies and organisations should strive to take part in international boards, panels and association to foster a culture of standardisation, interaction and cooperation.
4.6	Don't extend information rights law at this time - In the UK there is now a drive from the Information Commissioner to extend FOI - whilst access to public sector information is a good ideal this is not the time to tie up the public administrative sector and those in the private sector who engage in this in further change. It is time consuming to make change and accountability is not the UK's overarching priority. The French don't even have FOI! We need to make global partnerships for business and research purposes.
4.7	lobby government to clarify and provide sound foundation for skilled work visas, certainty of residence lobby government to continue cooperative funding arrangements
4.8	Better narratives from politicians to promote connection. Also more 'ethical' governance from social media. Social media was felt to be a good stimulus but now it feels like Facebook, Google and others are corrupt/unethical just in different ways. So this also does not promote positive channels for community cohesion and communication.
4.9	Stopping Brexit, ministers who pursue/d racist immigration policies to resign, and the press to stop stirring up racial and xenophobic hatred.

4.10	Difficult to say - would have to look into alternatives for university funding/business model. Research would need to look into cross working alternative structures and easy ability for EU HE staff to stay in UK
